# Assessing Lettuce Exposure to a Multipharmaceutical Mixture under Hydroponic Conditions: Findings through LC-ESI-TQ Analysis and Ecotoxicological Assessments

**DOI:** 10.1021/acsomega.4c08013

**Published:** 2024-11-29

**Authors:** Ludmila Mravcová, Vojtěch Jašek, Marie Hamplová, Jitka Navrkalová, Anna Amrichová, Helena Zlámalová Gargošová, Jan Fučík

**Affiliations:** † Institute of Chemistry and Technology of Environmental Protection, Faculty of Chemistry, Brno University of Technology, Purkyňova 118, 612 00 Brno, Czech Republic; ‡ Institute of Materials Chemistry, Faculty of Chemistry, Brno University of Technology, Purkyňova 118, 612 00 Brno, Czech Republic

## Abstract

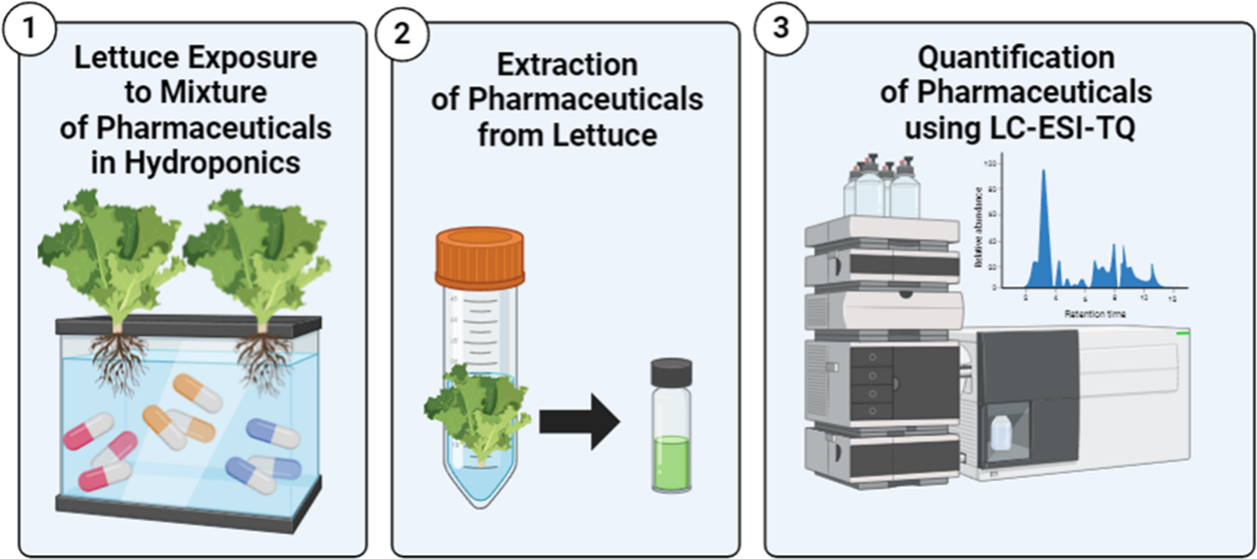

The escalating global water scarcity demands innovative solutions, one of which is hydroponic vegetable cultivation systems that increasingly use reclaimed wastewater. Nevertheless, even treated wastewater may still harbor various emerging organic contaminants, including pharmaceuticals. This study aimed to comprehensively assess the impact of pharmaceuticals, focusing on bioconcentration factors (BCFs), translocation factors (TFs), pharmaceutical persistence in aqueous environment, ecotoxicological end points, and associated environmental and health risks. Lettuce (*Lactuca sativa*) was cultivated hydroponically throughout its entire growth cycle, exposed to seven distinct concentration levels of contaminants ranging from 0 to 500 μg·L^–1^ over a 35-day period. The findings revealed a diverse range of BCFs (2.3 to 880 L·kg^–1^) and TFs (0.019–1.48), suggesting a high potential of pharmaceutical uptake and translocation by *L. sativa*. The degradation of 20 pharmaceuticals within the water-lettuce system followed first-order degradation kinetics. Substantial ecotoxicological effects on *L. sativa* were observed, including increased mortality, alterations in root morphology and length, and changes in biomass weight (*p* < 0.05). Furthermore, the estimated daily intake of pharmaceuticals through *L. sativa* consumption suggested considerable health risks, even if lettuce would be one of the many vegetables consumed. It is hypothetical, as the values were calculated. Moreover, this study assessed the environmental risk associated with the emergence of antimicrobial resistance (AMR) in aquatic environments, revealing a significantly high risk of AMR emergence. In conclusion, these findings emphasize the multifaceted challenges posed by pharmaceutical contamination in aquatic environments and the necessity of proactive measures to mitigate associated risks to both environmental and human health.

## 1 Introduction

As the global population continues to grow, challenges such as water scarcity, environmental pollution, and limited agricultural land persist.^1,2^ Currently, agriculture accounts for approximately 70% of freshwater consumption to meet escalating food demands, while 20% is in industry and 10% is allocated to domestic use.^1,3^ Therefore, current research endeavors are strongly focused on identifying alternative water sources, and agricultural practices, particularly to alleviate the pressure on freshwater reserves caused by agricultural activities.^1^

Global wastewater production annually reaches an estimated 1000 billion m^3^,^1^^1^ prompting the exploration of solutions such as reclaimed water use in hydroponics^4−7^ or aquaponics.^8^ Employing treated wastewater for irrigation not only conserves drinking water resources but also recycles nutrients for plant growth,^4,5^ thereby contributing to food security.^5^ Furthermore, hydroponic systems offer advantages such as accelerated growth, increased yields per unit area, enhanced water efficiency, reduced fertilizer usage, and improved nutritional quality, enabling year-round vegetable production^4,9,10^ and in the future could become a strategy for sustainably feeding the world’s growing population.^11^ Additionally, hydroponic plant cultivation can serve as an additional wastewater treatment step for enhanced nutrient and micropollutant removal,^4^ particularly when employed for treating domestic^6,7,12^ or municipal and industrial wastewater.^5,6,13^ In hydroponic systems, a diverse range of vegetables, including silverbeet, tomatoes, cucumbers, radishes, lettuce, spinach, beans, and herbs, are commonly cultivated, with lettuce being the most prevalent.^1,7,9,14^

However, even after treatment, wastewater may still harbor heavy metals, human pathogens, and emerging organic pollutants.^1,6,9^ Among these emerging pollutants are pharmaceuticals, which can be found in wastewater at concentrations ranging from hundredths of ng·L^–1^ to hundreds of μg·L^–1^.^15,16^ Consequently, these micropollutants have the potential to be absorbed by plants and may enter the food chain,^10^ or can also contribute to the spread of antimicrobial resistance within both environmental ecosystems and human populations.^17^

Recent studies have conducted hydroponics experiments using various vegetables, with lettuce being the most commonly studied.^18−22^ These investigations typically involve hydroponic experiments utilizing pharmaceutical mixtures containing either a single or a few drugs <6 PhACs,^18−20,23,24^ although some studies include up to 13 pharmaceuticals,^22^ and less frequently, up to 20 compounds.^18,21^ Hydroponic experiments are conducted in various ways: either by exposing pregrown seedlings for their entire growth period,^20,21^ exposing them only during a portion of the growth period,^18,23^ or by exposing fully grown lettuce to a pharmaceutically contaminated solution,^19,22^ with the former case reflecting more realistic conditions. Nutrient solutions are typically contaminated at environmentally relevant concentrations ranging from 0.3 to 100 μg·L^–1^,^18−22,24^ either at single^19,20,22^ or up to three pharmaceutical concentration levels within a single study.^18,21^ The objectives of these studies varied, aiming to determine bioconcentration factors (BCFs) and translocation factors (TFs), assess the dependence of intake on drug properties, or establish the risk of emerging contaminants on human health.^9,18,25,26^ Additionally, some investigations evaluated several ecotoxicological end points on *L. sativa* due to the presence of pharmaceuticals.^18,27^ Nevertheless, it is rare for a single study to evaluate all of these parameters, including ecotoxicological results.

Lettuce was selected for our hydroponic experiments as it is one of the most widely grown leafy vegetables in commercial settings,^28−30^ making it particularly susceptible to exposure if pharmaceutical-contaminated wastewater is used in such systems. The aim of this study was to determine bioconcentration factors (BCFs) and translocation factors (TFs) of *L. sativa* grown cultivated hydroponically throughout its entire growth cycle, while exposed to seven distinct concentration levels of contaminants, spanning from 0 to 500 μg·L^–1^. This concentration range encompassed both environmentally relevant contamination levels and those elevated by an order of magnitude. On the contrary to previous studies, BCFs and TFs were calculated for each sampling time using an innovative methodology that integrated time-weighted average aquatic concentrations for 20 pharmaceutical compounds, thereby providing a more accurate reflection of real-world scenarios. Sampling of lettuce roots, leaves, and hydroponic solutions took place on days 14, 21, 28, and 35, affording an objective view into pharmaceutical uptake, translocation, and persistence within aquatic ecosystems. In addition to employing liquid chromatography–mass spectrometry (LC-MS) analyses, the study also focused on various ecotoxicological end points, with particular emphasis on observable physiological effects such as mortality rate, root morphology alterations, and biomass weight of *L. sativa*. Statistical analyses were conducted to ascertain the significance of the findings. Furthermore, the novelty of this study lies in its extended scope, which both estimates the human daily intake resulting from *L. sativa* contamination and assesses the associated risk concerning the potential emergence of antimicrobial resistance in aquatic environments.

## 2 Materials and Methods

### 2.1 Chemicals and Standards

Ammonium nitrate (>99.5%), calcium nitrate tetrahydrate (>99%), citric acid monohydrate (≥99%), disodium hydrogen phosphate dodecahydrate (≥99%), ferrous sulfate heptahydrate (>99.7%), hydrochloric acid (35%), potassium nitrate (>99.9%), sodium sulfate anhydrous (≥95%), and sulfuric acid (≥96%) were purchased from Lach-ner, s.r.o. (Czech Republic). Acetonitrile (LC-MS grade), diethylenetriaminepentaacetic acid (≥99%), and methanol (LC-MS grade) were purchased from VWR (Czech Republic). Copper sulfate pentahydrate (>98%), formic acid (LC-MS grade), manganese sulfate monohydrate (>99%), sodium chloride (>99%), sodium molybdate dihydrate (>99.5%), and zinc sulfate heptahydrate (>99%) were purchased from Sigma-Aldrich (Germany). Sodium hydroxide (>98%) and potassium hydroxide (>98%) were purchased from Penta Chemicals (Czech Republic). Boric acid (>99.5%), magnesium sulfate heptahydrate (>99%), and potassium phosphate monobasic (>99.5%) were purchased from Lachema n.p. (Czech Republic).

The following pharmaceuticals were used: acebutolol hydrochloride (≥99%), naproxen (≥99%), and sulfacetamide (≥98%) were purchased from Honeywell. Ofloxacin (≥98%) was purchased from Thermo Fisher Scientific. Atenolol (≥98%), azithromycin (≥98%), ciprofloxacin (≥98%), clarithromycin (≥97%), enrofloxacin (≥99%), moxifloxacin (≥96%), nadolol (≥98%), norfloxacin (≥98%), pefloxacin mesylate dihydrate (≥97%), propranolol hydrochloride (≥98%), roxithromycin (≥95%), sulfadimethoxine (≥98%), sulfamethoxazole (≥98%), sulfapyridine (≥99%), tetracycline (≥98%), and trimethoprim (≥98%) were purchased from Sigma-Aldrich (Germany).

Nitrogen gas (4.7) and Argon gas (5.0) were purchased from SIAD Czech spol. s.r.o. (Czech Republic). Nylon syringe filters (13 mm, 0.22 μm) were purchased from Chromservis (Czech Republic). For QuEChERS, dispersive SPE (dSPE): DSC-18 SPE and PSA SPE were purchased from Sigma-Aldrich (Germany).

### 2.2 Uptake of Pharmaceuticals by Lettuce under Hydroponic Conditions

This study was conducted in a grow box (Green-Qube 1020L) under controlled conditions with a 16 h photoperiod (17,500 lx; LED panel: ViparSpectra XS2000 230W), air temperature of 23 ± 1 °C, and air humidity of 45 ± 5%. A single extraction fan and two oscillating fans were placed in the grow box to ensure the appropriate air exchange and flow. For hydroponic experiments, the deep water culture (DWC) method was used to grow lettuce. Aquariums (20 × 20 × 20 cm^3^) wrapped with dark duct tape were used as containers. To prepare hydroponic solutions, salts according to modified Sonneveld′s recipe for lettuce were dissolved in deionized water^31^ as detailed in Tables S1 and S2. Subsequently, the water was spiked with a mixture of 20 pharmaceuticals (physicochemical properties of PhACs in Table S3) at 7 concentration levels ranging from 0 to 500 μg·L^–1^ (specifically 0; 5; 10; 25; 50; 100; 500 μg·L^–1^). Seeds of lettuce (*L. sativa*, Australischer Gelber) were purchased from FloraSelf (Czech Republic). Subsequently, eight sprouted lettuces (5 days old) per aquarium were planted into rock wool and hydroponic cups and 4 replicates of each experiment were prepared. Electrical conductivity (EC) during the experiment was adjusted in the following way: 0.95–1.05 mS·cm^–1^ up to the 14th day, 1.3–1.5 mS·cm^–1^ up to the 21st day, 1.8–1.9 mS·cm^–1^ up to 28th day, and 2.2–2.3 mS·cm^–1^ up to 35th day. Meanwhile, the pH value was kept constant at 6.3 ± 0.1 for the entire duration of the experiment. Both the EC and pH were adjusted daily within the appropriate range. The amount of water was refilled daily to keep the water volume constant for the uptake experiments. The aquariums were randomly placed in the grow box, and the aquarium positions were changed every third day to compensate for differences in light intensity. The aquariums were aerated for 10 min every hour. The lettuces were sampled after 14; 21; 28 and 35 days of exposure for both control and contaminated samples. Images of *L. sativa* during its growth in a hydroponic solution are available in Figures S1–S4. Lettuce samples were obtained by sampling five lettuces from each aquarium after 14 days and consequently by sampling a single lettuce every week. The obtained samples of leaves and roots were washed with deionized water to remove pharmaceuticals from the surface. Lettuce samples from day 14 were analyzed as whole plants because of their low biomass. Samples from subsequent days were separated into roots and leaves for individual analysis. Additionally, at a concentration of 500 μg·L^–1^, only the lettuce samples from day 14 were analyzed due to the extremely high mortality rate, as discussed in the ecotoxicological Section 3.5. Water for PhACs quantification was sampled at regular intervals over a 35-day exposure experiment. Immediately after sampling the lettuce, ecotoxicological end points were evaluated, including mortality rate, root biomass weight, leaf biomass weight, and root morphology.

### 2.3 Extraction and Quantification of Pharmaceuticals from Lettuce and Water Samples

We utilized the QuEChERS-based extraction method developed and validated by ref (32) for the quantification of PhACs in lettuce leaves and lettuce roots. Detailed protocols for the QuEChERS method can also be found in the Supporting Information, Appendix 1. The resulting lettuce extracts were analyzed using the LC-MS/MS method, which is outlined in the same publication^32^ and further detailed in the Supporting Information, Appendix 2. The same LC-MS/MS method was employed for analyzing hydroponic water samples. These samples required no preparation beyond filtration through 0.22 μm nylon syringe filters (13 mm diameter) before being transferred to 2 mL glass vials.

## 3 Results and Discussion

### 3.1 Lettuce Exposure to Different Concentrations of Pharmaceuticals under Hydroponics Conditions

**Figure 1:**
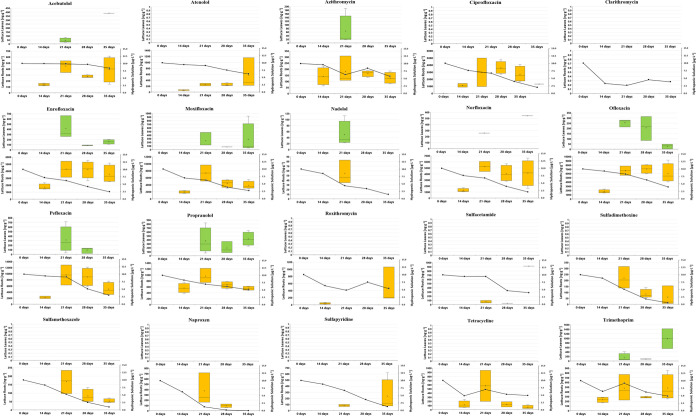
Dynamics of individual PhAC concentrations in lettuce leaves (top, green box plot) and roots (bottom, orange box plot) in [ng·g^–1^ dw], alongside concentrations in hydroponic solution (bottom graph, right *Y*-axis in μg·L^–1^) over 35 days, with initial exposure concentration of 10 μg·L^–1^.

**Figure 2:**
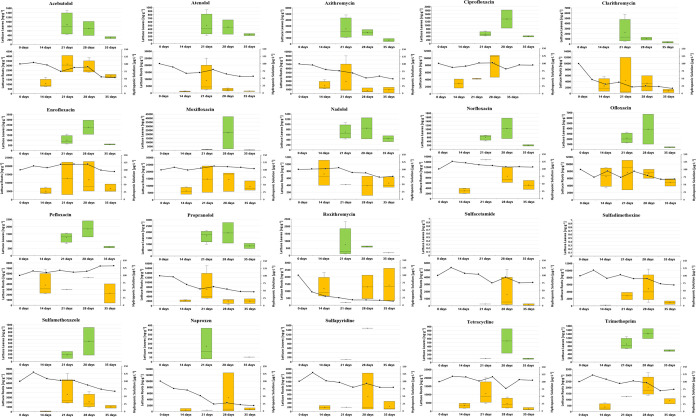
Dynamics of individual PhAC concentrations in lettuce leaves (top, green box plot) and roots (bottom, orange box plot) in [ng·g^–1^ dw], alongside concentrations in hydroponic solution (bottom graph, right *Y*-axis in μg·L^–1^) over 35 days, with initial exposure concentration of 100 μg·L^–1^.

*L. sativa* seedlings were exposed to a mixture of 20-PhACs representing various therapeutic classes. To thoroughly evaluate both bioconcentration factors (BCFs) and translocation factors (TFs), the experiments involved a wide range of aquatic contamination with concentrations ranging from 0 to 500 μg·L^–1^ and samples were collected on days 14, 21, 28, and 35. In addition to determining BCFs and TFs (Table 1)the measured concentrations of PhACs in the water-plant system (including concentrations in roots, leaves, and hydroponic solution) are displayed in Figures 1 and 2, which correspond to concentrations of 10 and 100 μg·L^–1^, respectively. Furthermore, PhAC concentrations in the roots and leaves of *L. sativa* across all concentration levels and time points are presented in heatmaps in Figures S5 and S9. Similar to other studies,^19,20^ whole lettuce-water-pharmaceutical system was analyzed at regular intervals, whereas many studies have only analyzed concentrations at a single sampling time.^18,21,24^

Moreover, principal component analysis (PCA) was conducted to evaluate variability and identify patterns in the concentrations of pharmaceutical residues in *L. sativa* samples grown hydroponically. The analysis focused on comparing residue levels in roots and leaves across different exposure times (Figure S10). The two principal components, PC1 and PC2, explain 35.5 and 14.6% of the variance in the data, respectively, indicating that these two components capture approximately half of the data set’s total variability. The PCA plot color-codes the sample types according to tissue and exposure time: leaf samples are represented in green, young *L. sativa* seedlings (14 days old, analyzed as whole plants with both roots and leaves) are shown in black, while root samples transition from light orange for 21 days to orange for 28 days and finally to red for 35 days. Leaf samples cluster closely, while young seedling samples are interspersed between leaf and root samples, suggesting statistically significant distinctions among sample types. Root samples, particularly at longer exposure times (28 and 35 days), show higher concentrations of pharmaceutical residues, as indicated by their distinction along the principal components. Different pharmaceutical groups, such as fluoroquinolones (FQs), sulfonamides (SAs), β-blockers (BBs), and macrolides (MLs), exhibit varying loadings along PC1 and PC2, indicating that these compounds contribute differently to the variance observed in the data. However, pharmaceuticals from the same class, such as SAs and FQs, tend to exhibit comparable loadings along the axes, suggesting that these compounds may have analogous uptake or metabolic behaviors in lettuce.

### 3.2 Calculation of Bioconcentration and Translocation Factors

Bioconcentration factors (BCFs) within the plant-water system (or alternatively in plant–soil) are commonly calculated using eq 1, which is usually derived from either initial aquatic (or soil) exposure concentration^33−35^ or aquatic (or soil) concentration at the time of sampling.^36^ However, relying solely on either initial or final concentrations can result in inaccurate estimations of BCFs, because these methods do not account for the degradation of PhACs over time. Degradation rates (*k* [d^–1^]) and their standard errors (SE) for each substance in the lettuce-water system were derived via linear regression using eq 2, and the results are detailed in Table 1. Consistent with previous studies,^37−39^ PhAC degradation in aquatic environments adhered to first-order kinetic models. Meanwhile, in the study,^21^ the degradation of PhACs in the hydroponic system was deemed negligible due to processes such as photolysis, abiotic transformation, or microbial transformation, although these processes are present within real-world conditions.

**Table 1:** Results of Pharmaceutical Uptake by *L. sativa* under Hydroponic Conditions^a^

		bioconcentration factors at various sampling days								translocation factors at various sampling days						degradation kinetics		
pharmaceutical group	pharmaceutical name	BCF_14D_ [L·kg^–1^]	SE of BCF_14D_ [L·kg^–1^]	BCF_21D_ [L·kg^–1^]	SE of BCF_21D_ [L·kg^–1^]	BCF_28D_ [L·kg^–1^]	SE of BCF_28D_ [L·kg^–1^]	BCF_35D_ [L·kg^–1^]	SE of BCF_35D_ [L·kg^–1^]	TF_21D_ [−]	SE of TF_21D_ [−]	TF_28D_ [−]	SE of TF_28D_ [−]	TF_35D_ [−]	SE of TF_35D_ [−]	*k* [d^–1^]	*k* [d^–1^]	linearity range [μg·L^–1^]
β-blockers	acetobutolol	21.4	1.6	65	4	61	4	35	3	0.082	0.011	0.119	0.022	0.120	0.004	0.0061	0.0011	up to 50
	atenolol	29	5	230	40	78	4	53	4	0.1280	0.0015	0.068	0.003	0.062	0.006	0.019	0.003	up to 50
	nadolol	26	4	5.4	0.7	5.7	0.7	6.6	2.2	1.48	0.06	1.02	0.03	N.D.		0.037	0.004	up to 50
	propranolol	55	8	185	12	45	4	64	8	0.37	0.07	0.63	0.05	0.31	0.04	0.0179	0.0018	up to 50
fluoroquinolones	ciprofloxacin	82	6	880	130	280	30	201	12	0.024	0.005	0.085	0.020	0.144	0.022	0.034	0.004	up to 50
	enrofloxacin	126	10	1140	100	491	11	780	100	0.032	0.009	0.048	0.004	0.055	0.003	0.029	0.003	up to 50
	moxifloxacin	290	30	1590	90	462	18	357	16	0.030	0.004	0.0285	0.0023	0.050	0.006	0.025	0.003	up to 50
	norfloxacin	147	7	1160	140	350	30	518	19	0.0291	0.0008	0.050	0.011	0.019	0.004	0.033	0.004	up to 50
	ofloxacin	163	16	800	180	740	40	470	50	0.063	0.008	0.0313	0.0021	0.053	0.005	0.017	0.003	up to 50
	pefloxacin	292	24	N.D.		640	110	680	40	0.0312	0.0019	0.039	0.007	0.036	0.011	0.022	0.006	up to 50
macrolides	azithromycin	40	4	73	10	13.5	0.5	19	3	0.95	0.08	N.D.		0.42	0.07	0.0154	0.0023	up to 50
	clarithromycin	61.5	0.5	N.D.		88	7	29.9	2.4	N.D.		N.D.		0.419	0.015	0.032	0.004	up to 50
	roxithromycin	28	4	N.D.		30.1	1.3	34	3	N.D.		0.110	0.021	N.D.		0.020	0.003	up to 50
NSAIDs	naproxen	37	3	200	40	210	30	16	5	0.503	0.012	0.043	0.004	N.D.		0.083	0.016	up to 50
sulfonamides	sulfacetamide	N.D.		N.D.		42	9	4.67	0.1	N.D.		0.051	0.010	N.D.		0.036	0.006	up to 50
	sulfadimethoxine	9.9	0.6	97	14	220	30	29.7	2.5	N.D.		0.040	0.004	N.D.		0.069	0.008	up to 50
	sulfamethoxazole	2.3	0.3	120	15	76	10	46.4	1.4	N.D.		0.21	0.04	N.D.		0.049	0.005	up to 50
	sulfapyridine	5.5	0.9	N.D.		15.1	1.7	9.83	0.2	N.D.		0.31	0.03	N.D.		0.045	0.005	up to 50
	trimethoprim	29.1	2.4	84	11	50	3	31	5	0.40	0.08	0.19	0.04	0.35	0.06	0.0160	0.0022	up to 50
tetracyclines	tetracycline	78	11	358	15	153	22	52	16	0.0706	0.0020	N.D.		0.089	0.010	0.0158	0.0019	up to 50

a Bioconcentration factors (BCFs), translocation factor (TF), degradation rate kinetics (k), and their respective standard errors (SE) for individual pharmaceuticals at different sampling days

To determine more confident BCFs, time-weighted average (TWA) aquatic concentrations were determined using eq 3. These TWA concentrations were further inserted into eq 1 for the calculation of BCF values (Table 1), which were determined as the slope of the linear equation *y* = *ax* (where *y* is the root concentration, *x* is the TWA aquatic concentration, and a (slope) represents BCF). This strategy of using time-weighted average concentration has also been suggested in some previous research studies^40,41^ but has not yet been adopted for BCF determination in the lettuce-water system. By incorporating the time variable in the determination, this method provides a more sophisticated and good measure, especially when the concentrations decrease over time. By accounting for variations in concentration over the exposure period, this method gives more weight to periods when compound levels are higher and sustained for longer durations, resulting in a more accurate average.

Similar to BCFs, translocation factors (TFs) were determined using the slope of a linear equation *y = ax* (where *y* represents the concentration in *L. sativa* leaves, *x* represents the concentration in *L. sativa* roots, and *a* (the slope) corresponds to the TF), as outlined in eq 4 and studies.^42,43^
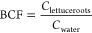
1where *C*_lettuceroots_ [ng·g^–1^] stands for the concentration in lettuce roots grown under hydroponic conditions at the end of the exposure, while *C*_water_ [ng·L^–1^] indicates the concentration of pharmaceuticals in the hydroponic solution, which could be the initial concentration, the concentration at the experiment’s conclusion, or the time-weighted average concentration.
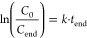
2where *C*_0_ [ng·L^–1^] represents the initial pharmaceutical concentration in aqueous hydroponic solution, *C*_end_ [ng·L^–1^] is the concentration of pharmaceutical at the conclusion of the experiment, *k* [d^–1^] indicates the degradation rate kinetics of pharmaceutical, and *t*_end_ [day] stands for the duration of the experiment.
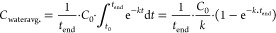
3where *C*_water,avg._ [ng·L^–1^] denotes the time-weighted average pharmaceutical concentration in hydroponic solution, *t*_end_ [d] stands for the duration of the experiment, *C*_0_ [ng·L^–1^] stands for the initial pharmaceutical concentration in hydroponic solution, and *k* [d^–1^] stands for the degradation rate kinetics of pharmaceutical.
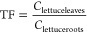
4where *C*_lettuceleaves_ [ng·g^–1^] stands for the concentration in lettuce leaves at the end of the experiment and *C*_lettuceroots_ [ng·g^–1^] stands for the concentration in lettuce roots at the end of the experiment.

### 3.3 Bioconcentration Factors

A bioconcentration factor greater than 1 indicates significant accumulation of the pharmaceutical in *L. sativa* roots, whereas a bioconcentration factor in the range of (0; 1) suggests that uptake occurs, albeit not at a significant rate. In agreement with previous studies,^42,43^ the BCFs were well modeled using linear equations, although this was only observed within the concentration range of 0–50 μg·L^–1^. This linearity indicates that the PhAC concentration in *L. sativa* roots is proportional to the PhAC concentration in the hydroponic solution. The limited range of linearity could be attributed to several factors, such as increased metabolism rates at higher concentrations, limitations in the linear model, inaccuracies in measuring high concentrations, and inherent biological variability in *L. sativa*.

BCF values (Table 1) were determined for each sampling day at 14, 21, 28, and 35 days, and all estimated BCFs significantly exceeded the value of 1. Specifically, the results for different therapeutic classes of PhACs were as follows: for β-blockers (BBs): 5.4 to 230 L·kg^–1^; for fluoroquinolones(FQs): 82 to 1590 L·kg^–1^; for macrolides (MLs): 13.5 to 88 L·kg^–1^; for NSAIDs: 16 to 210 L·kg^–1^; for sulfonamides (SAs): 2.3 to 220 L·kg^–1^; and for tetracyclines (TCs): 52 to 358 L·kg^–1^. Additionally, a *t* test was conducted to assess whether the BCF for each drug varied significantly, depending on the sampling time. According to Table S5, the majority of BCFs (when BCF values were determined) differed across sampling times, despite PhAC concentrations in *L. sativa* being directly proportional to aquatic concentrations at specific sampling times. Different BCF values across sampling days can be explained by several factors: the dilution effect due to *L. sativa* growth, varying plant metabolism across its lifespan, differing TWA aquatic concentrations resulting from PhAC degradation, and biological variability in *L. sativa*’s absorption and accumulation processes over its life cycle.

The highest average BCFs for all PhAC groups were observed on day 21, whereas the lowest averages were observed on either day 14 (for SAs, FQs, and BBs) or day 35 (for TCs, NSAIDs, and MLs). Comparing BCFs across individual groups, the highest overall averages were as follows: FQs (437 L·kg^–1^) > TCs (160 L·kg^–1^) > NSAIDs (116 L·kg^–1^) > BBs (60 L·kg^–1^) > SAs (51 L·kg^–1^) > MLs (42 L·kg^–1^). These values differed by orders of magnitude in some cases (e.g., FQs versus other therapeutic classes), although in certain instances only one drug within a PhAC group was evaluated (e.g., NSAIDs and TCs). These differences in uptake rates by *L. sativa* can be attributed to various PhAC properties, such as molecular weight, p*K*_a_ values, log *P* (or log *D*_ow_ for ionizable drugs), number of hydrogen bonds, and water solubility.^25,26^ Additionally, environmental factors such as pH of water environment, presence of other dissolved chemicals, photoperiod, and air temperature and humidity play significant roles.^25,26^ Studies^26,42,44^ have shown that uptake rates can vary among different plant species, cultivars, or genotypes^34,45^ due to differences in plant physiology, root morphology, and other biological characteristics such as lipid and carbohydrate content.

Compared with soil environments, hydroponic systems are significantly simpler, lacking soil particles, high levels of divalent cations (e.g., Ca^2+^, Mg^2+^), organic matter (such as humic and fulvic acids), and clay mineral fractions. As a result, adsorption is less significant, enabling the modeling of pharmaceutical uptake by plants based on their properties.^22,25^ Moreover, controlling the composition of the hydroponic solution is much easier compared with soil properties, allowing for more precise experimental conditions.^23^ The differences mentioned above are reflected in the fact that under hydroponic conditions, the uptake rate is significantly higher (often reported BCFs ≫ 1^21^) than in a soil environment (often reported BCFs < 1^34,35^ and in some cases BCFs > 1^46^). However, comparing BCFs in the two environments can be challenging. In soil, BCFs are typically expressed as dimensionless units [-],^35^ whereas in aquatic environments, they are reported in units of [L·kg^–1^; mL·g^–1^; or L·g^–1^].^21,22,24^

Generally, studies^18,19,21,22^ have reported significantly higher concentrations of PhAC residues in the root parts of plants. Specifically, ref (18) investigated hydroponically grown *L. sativa* exposed to concentrations of 1, 10, and 100 μg·L^–1^, determining BCFs as the mean across 10 and 100 μg·L^–1^. Contrary to our results, this study reported BCFs < 1 for all tested PhACs, specifically nevirapine, lamivudine, efavirenz, and oseltamivir. This discrepancy could be explained by the fact that these compounds are antivirals and antiretrovirals, which were therapeutic groups not investigated in our study. In agreement with our results, ref (21) determined BCFs as the average of *L. sativa* exposure to 0.5 and 5 μg·L^–1^ for 16 PhACs, with values ranging from 10^–1^ to 10^3^, depending on the specific PhAC. Moreover, in agreement with our results, ref (22) reported that BCFs can differ depending on the sampling time, either significantly or not, depending on the pharmaceutical. Additionally, ref (24) reported that PhAC concentrations within the plants did not significantly differ between treatments when exposed to a single pharmaceutical alone or in binary/tertiary combinations.

Furthermore, we investigated correlations between BCFs and PhAC properties, but no significant correlations were found for either the molecular weight or log *P* (log *D*_ow_). Similarly, several studies^20,22,25^ have attempted to model uptake rates based on PhAC properties. However, these studies present conflicting results; while some studies^21,26,42^ reported dependencies of log BCF on log *D*_ow_, others^20−22,25^ observed no correlation. A similar situation was also noted with the molecular weight. Nevertheless, these studies agreed on a dependence of log BCF on log *K*_ow_ for only neutral compounds. Such discrepancies may be attributed to variations in the experimental design and the conditions. Additionally, ref (20) highlighted that plants exude organic and inorganic acids through their roots, altering the pH of the medium near the root surface (1–2 mm) over time. This phenomenon could elucidate the lack of correlation between Dow and analyte depletion from the culture medium as differences in medium pH lead to inaccurate Dow calculations.

### 3.4 Translocation Factors

PhACs with a translocation factor (TF) of less than 1 are primarily retained in the roots, suggesting limited transfer to the leaves. Conversely, a TF greater than 1 indicates effective translocation from roots to leaves, highlighting a greater potential for bioconcentration in the aboveground parts of *L. sativa*.^18,21^ The determined TFs for the individual sampling times of 21; 28; and 35 are displayed in Table 1, ranging from 0.019 to 1.48. The TF was not calculated for day 14, as the entire *L. sativa* plant was analyzed as a single sample without separation of the roots and leaves.

Specifically, the results for different therapeutic classes of PhACs were as follows: for β-blockers: 0.062–1.48; for fluoroquinolones: 0.019 to 0.144; for macrolides: 0.11 to 0.95; for NSAIDs: 0.043 to 0.503; for sulfonamides: 0.01 to 0.4; and for tetracyclines: 0.0706 to 0.089. In addition, a *t* test was conducted to assess whether the TF for each drug varied significantly, depending on the sampling time. According to Table S5, for most PhACs, TFs did not differ significantly across sampling times (when TF values were determined), as PhAC concentrations in *L. sativa* leaves are directly proportional to concentrations within the roots. Small differences across sampling times can be attributed to factors such as varying plant metabolism across its lifespan and biological variability in *L. sativa’*s translocation processes over its life cycle. Moreover, with the exception of nadolol (β-blocker), TFs did not exceed a value of 1, suggesting that translocation to the aerial part of *L. sativa* is not efficient.

Reference (18) investigated hydroponically grown *L. sativa* exposed to concentrations of 1, 10, and 100 μg·L^–1^, determining TFs as a mean across 10 and 100 μg·L^–1^. Consistent with our results, this study found that TF values are typically <1, except for nevirapine, which had a TF > 2. Similar findings were reported by studies,^19,21^ noting that the majority of pharmaceutical residues were found in the roots of *L. sativa*. Furthermore, ref (21) reported TFs for various hydroponically grown vegetables, including *L. sativa*, at concentrations of 0.5 and 5 μg·L^–1^. This study documented TFs for 16 pharmaceuticals as average across the two spike levels, ranging from 10^–2^ to 10^2^, depending on the specific pharmaceutical. Previous studies have generally reported that translocation in plants depends on various factors, including plant type and physiology,^19,21,26,42,44^ the physicochemical properties of PhACs,^26^ the surrounding environment and its impact on plant metabolism,^26^ and the concentration of pollutants in the environment and plant roots.^43^

### 3.5 Ecotoxicological End Points

Earlier ecotoxicological studies have shown that phytotoxicity depends on the type, mixture, and concentration of PhACs, as well as plant species and experimental conditions such as exposure duration and method.^27,47^ This phytotoxicity manifests as lowered germination rates, increased mortality rate, reduced biomass, stunted shoot and leaf development,^18,27^ and other effects such as necrotic spots, morphological deformations, burnt edges, yellowish patches, reduced photosynthetic pigments, decreased evapotranspiration, and reactive oxygen species accumulation.^27^ PhACs can also target ion channels and enzymes, inhibiting the transport of essential elements needed for plant growth.^27^ Additionally, studies have reported significant effects on the proteome^48^ and lipidome^49^ of *L. sativa* grown under hydroponic conditions.

**Figure 3:**
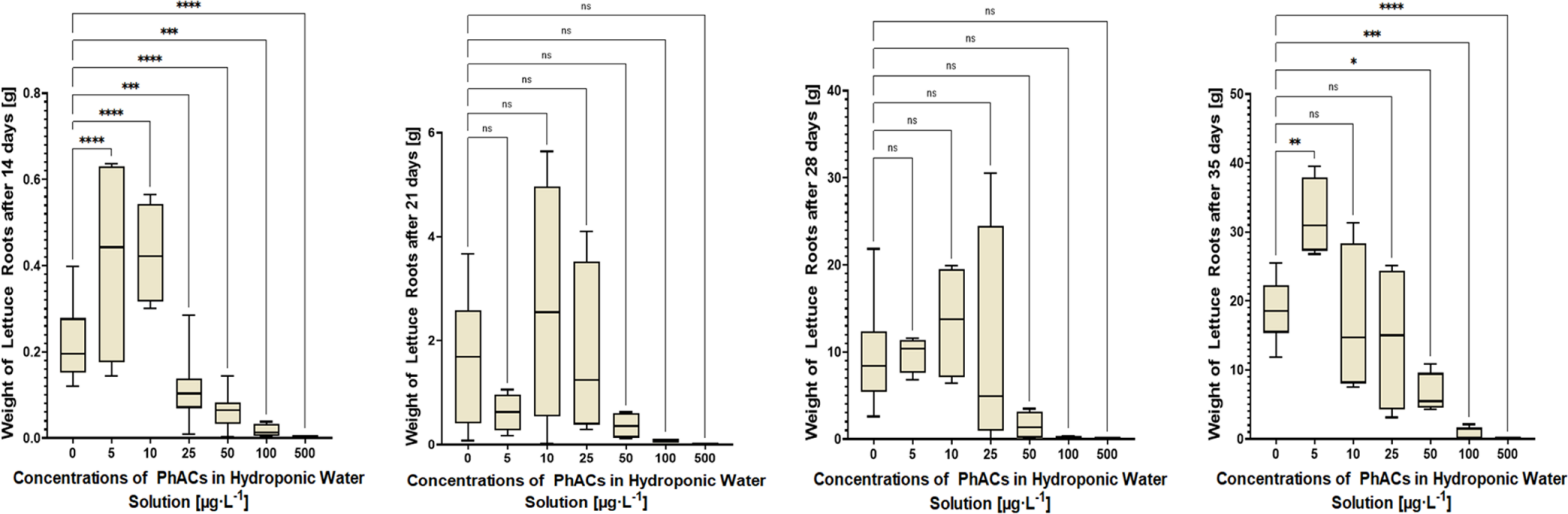
Weight of lettuce roots at different pharmaceutical concentrations on sampling days 14, 21, 28, and 35.

**Figure 4:**
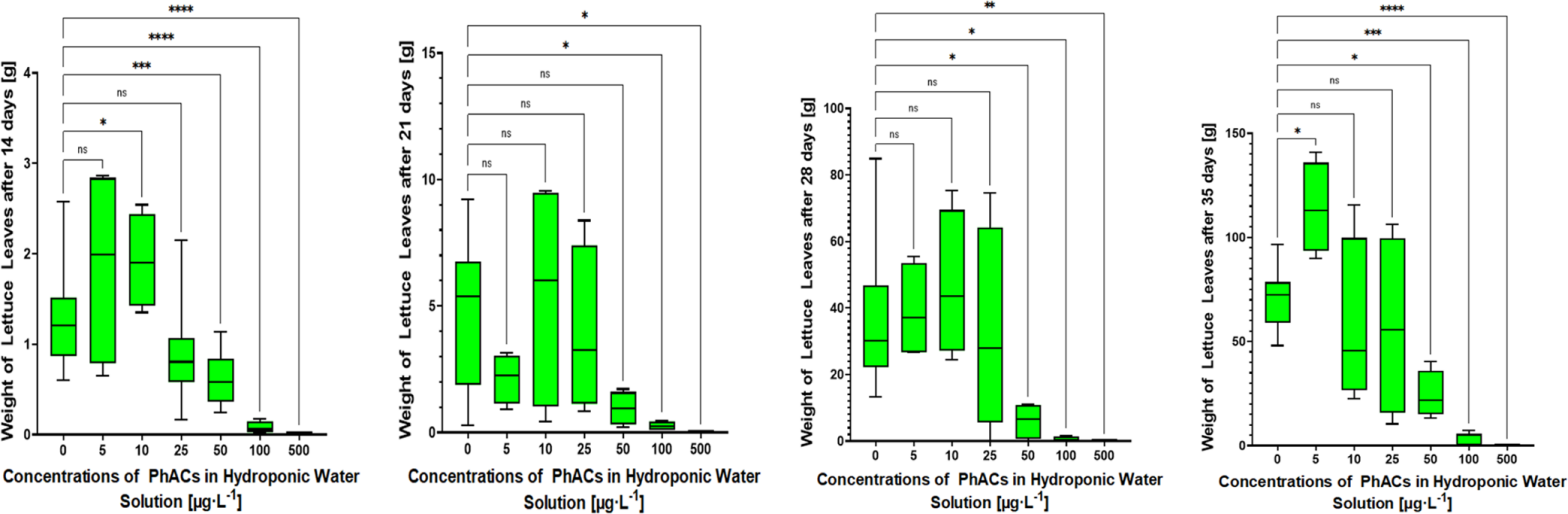
Weight of lettuce leaves at different pharmaceutical concentrations on sampling days 14, 21, 28, and 35.

In our study, we focused on the visible physiological effects such as mortality rate, weight, and length of *L. sativa* roots, root morphology, and weight of leaves. *L. sativa* was cultivated under hydroponic conditions and exposed to a PhAC mixture at concentrations from 0 to 500 μg·L^–1^. Within the concentration range of up to 50 μg·L^–1^, no significant increase in the mortality rate after 14 days was observed (*p* > 0.05). However, significantly increased mortality rates were observed at concentrations of 100 and 500 μg·L^–1^ (*p* < 0.01), with mortality rates of 25 and 75%, respectively. Moreover, the weight (Figure 3) and length (Figure S11) of lettuce roots were statistically evaluated on days 14; 21; 28; and 35. Consistent with previous studies,^18,50^ a nonlinear dose–response relationship known as hormesis was observed. At low concentrations, increased root weight and length were noted, whereas higher concentrations led to decreased root weight and length. These findings are supported by Figure S12, which shows a significantly altered root morphology of *L. sativa*, characterized by a filamentous structure and the absence of discernible small lateral roots at concentrations greater than 50 μg·L^–1^. This phenomenon indicates phytotoxicity and aligns with the concept of physiological perturbation or stress within the plant system. Exposure to PhACs can induce a spectrum of responses in plants, including a reduction in lateral root development and alterations in the architectural dynamics of the root system, as exemplified by the filamentous growth pattern observed in Figure S12. Additionally, the weight of lettuce leaves (Figure 4) was evaluated at days 14, 21, 28, and 35. Similar to the roots of *L. sativa*, a hormesis effect was observed in the leaves. The impact of the PhAC mixture on lettuce biomass diminished at days 28 and 35, likely due to the degradation of PhACs, the growth of *L. sativa*, or a combination of both factors. Despite decreasing concentrations of PhACs (as shown in Figures 1 and 2), known and unknown degradation products and metabolites can form,^23,51,52^ potentially decreasing or increasing phytotoxicity as their structures and toxicities are not described.

Furthermore, partial least-squares–discriminant analysis (PLS-DA) using Metabo Analyst^53^ with the above-mentioned ecotoxicological end points was conducted (Figure S13). PLS-DA shows a clear distinction from the control group only in the case of concentrations of 50 and 100 μg·L^–1^ and also illustrates the Variable Importance in Projection (VIP) scores for various growth parameters of *L. sativa* under different concentrations of PhACs. The highest VIP scores were noted for leaf weight at 35 days, indicating its significant influence on the model. The plot clearly demonstrates a hormesis effect, where low concentrations of PhACs enhance root and leaf growth, as evidenced by increased weights and lengths at lower doses. In addition, at higher concentrations (≥50 μg·L^–1^), there is a marked increase in mortality rate and significant alterations in root morphology, as already described in previous paragraphs. These observations suggest phytotoxicity and physiological stress in *L. sativa* when exposed to PhAC contamination.

In agreement with our results, ref (18) reported hormesis at concentrations of 1 and 10 μg·L^–1^ and a significant reduction in leaf biomass at 100 μg·L^–1^. Although contrary to our results, the same study^18^ reported that leaf inhibition was less impacted compared with the root; this difference could be attributed to the different PhAC mixtures used. Moreover, ref (47) exposed *L. sativa* under hydroponic conditions to 0, 5, and 10 mg·L^–1^ of enrofloxacin, oxytetracycline, and tylosin. It reported that enrofloxacin and oxytetracycline reduced lettuce growth by 70%, whereas tylosin had no significant effect, confirming that the impact depends on the pharmaceutical and its therapeutic class. Reference (54) delved into the impact of carbamazepine (CBZ) on lettuce plants cultivated hydroponically, with findings suggesting a more sensitive response than merely observing changes in biomass weight or the root length. CBZ was detected in both roots and leaves, triggering oxidative stress by elevating hydrogen peroxide levels in both plant parts and malondialdehyde levels, specifically in leaves. Vital antioxidative enzymes such as SOD, CAT, GPOD, and APX played pivotal roles in alleviating oxidative stress in leaves, whereas CAT, GPOD, and GR were active in roots. Ascorbate and glutathione have emerged as crucial antioxidants in combating CBZ-induced stress. Remarkably, despite the roots being in direct contact with CBZ, the leaves manifested a more pronounced oxidative response. These insights underscore the need for a nuanced understanding beyond traditional biomarker assessments when evaluating the impact of contaminants such as CBZ on plant physiology.

### 3.6 Potential Health Risk and Risk of Antimicrobial Resistance

Following the approach used in previous studies,^55,56^ we applied Monte Carlo simulation to assess potential health risks associated with the intake of pharmaceutical residues present in 35-day-old lettuce, given its relevance as a commonly consumed leafy vegetable. The methodology for calculating the health hazard index (HI) is detailed in Supporting Information, Appendix 3, and is based on techniques established in earlier research.^26,55−57^ The HI is calculated as the sum of risk quotients (RQs) for each pharmaceutical compound, with thresholds of 0.01 and 0.05 considered benchmarks for notable and distinct human health risk, respectively.^26^ Using Monte Carlo simulation, we generated probability distributions for risk quotients and hazard index values (Table S7 and Figures S14 and S15), capturing the range and likelihood of potential health outcomes associated with pharmaceutical concentrations of 10 and 50 μg·L^–1^. These concentrations fall within the linear range of the determined bioconcentration factors (BCFs) and translocation factors (TFs), thus reflecting environmentally relevant exposure scenarios for the human consumption of leafy vegetables.

In contrast to previous studies on soil- and hydroponically grown lettuce,^2,21,58,59^ our findings, based on Monte Carlo simulation, indicate that the estimated HI values at a concentration of 10 μg·L^–1^ have a 66.18% probability of exceeding the threshold of 0.01 (Figure S14), signifying a considerable human health risk associated with long-term intake. At a concentration of 50 μg·L^–1^, there is a 66.51% probability that the HI will exceed the threshold of 0.05 (Figure S15), suggesting a distinct health risk. Although risk quotients (RQs) could not be determined for all 20 pharmaceuticals (PhACs) due to incomplete data on translocation factors, it is important to emphasize that our assessment focused exclusively on the HI for PhAC intake through lettuce consumption. However, lettuce represents only one among many commonly consumed vegetables, implying that similar contamination levels could exist in other vegetables grown in contaminated water. Furthermore, real-world scenarios typically entail wastewater contamination with a wider range of PhACs, along with their metabolites and degradation products, a significant portion of which may remain unidentified. These findings underscore an alarming potential for pharmaceutical contamination in commonly consumed vegetables, particularly lettuce grown hydroponically, to pose significant health risks through long-term exposure. However, as these results were obtained using hydroponic solutions, they may not perfectly reflect real-world conditions. This highlights the need for further investigation into the broader impact of wastewater contamination on edible plants and the importance of monitoring PhACs and their metabolites in agricultural water sources under varied growing conditions.

Furthermore, the consumption of fresh vegetables like lettuce without cooking, aimed at preserving their nutritional value, may inadvertently contribute to the transfer of antibiotic-resistant genes in the human gastrointestinal tract, posing a significant threat to public health.^17,60^ According to statistical estimates from ref (61), antimicrobial resistance (AMR) contributes to approximately 1.17 million deaths globally, with an additional 2.62 to 4.78 million deaths associated with AMR-related complications. To comprehensively assess the risks associated with AMR in the aquatic environment, we evaluated RQs at day 0 and after 35 days for concentrations of 10 and 50 μg·L^–1^, considering the degradation kinetics of PhACs. RQ values were calculated as the ratio of the measured environmental concentration (MEC) to a predicted no-effect concentration (PNEC), with detailed calculations outlined in Supporting Information, Appendix 4. The calculated RQs and the summation of RQs (∑RQs) are provided in Table S8. The interpretation of RQs followed commonly used thresholds: RQ values below 0.1 indicate low risk, values between 0.1 and 1 signify medium risk, and values exceeding 1 represent high risk.^62,63^Table S8 illustrates that RQs for both concentrations exceeded the threshold for high risk toward AMR by hundreds of times, as available PNEC-MIC (Predicted No-Effect Concentration—Minimum Inhibitory Concentration values for water environment) values ranged from tens to units of μg·L^–1^. Although RQ values have shown a gradual decrease over time due to antibiotic degradation, the formation of degradation products or metabolites was not considered during the determination of RQs.

These findings align with previous studies,^64,65^ emphasizing the significant environmental and health concerns associated with the emergence and spread of AMR across animal, human populations and environment. This highlights the urgent need to implement appropriate legislation regulating permissible residues of PhACs in both vegetables and wastewater. Furthermore, this emphasizes the importance of exploring and implementing tertiary treatment processes at wastewater treatment plants such as advanced oxidation processes, membrane filtration, and adsorption. These processes are vital for efficiently eliminating both organic micropollutants and resistant bacteria, thereby mitigating the risks posed by AMR to public health and the environment.

## 4 Conclusions

BCF and TF values were calculated for 20 pharmaceuticals, including antibiotics commonly found in wastewater. A unique method was employed to calculate BCFs and TFs, utilizing time-weighted average (TWA) concentrations in aquatic environments. This method considers both the initial concentration in the hydroponic solution and the degradation kinetics of PhACs. We conducted an ecotoxicological test with a focus on the visible physiological effects, such as mortality rate, weight, and length of *L. sativa* roots, root morphology, and leaf weight. Consequently, statistical analysis showed a significant effect of PhACs on all evaluated ecotoxicological end points (*p* < 0.05).

Furthermore, using Monte Carlo simulation, we estimated the daily intake of pharmaceutical residues in lettuce, indicating considerable human health risks, even when only lettuce leaves were contaminated and consumed. However, lettuce is only one of the many sources of vegetables that can be grown hydroponically. Under real conditions, it is likely that the other foodstuffs would also be contaminated, with the spectrum of organic pollutants being several times higher, suggesting an even more alarming state. We also assessed the potential environmental threat posed by antimicrobial resistance in aquatic environments, where the presence of ∑RQs raises significant concerns regarding the occurrence and spread of AMR among human, animal, and ecological communities. These findings highlight the urgent need to develop advanced wastewater treatment technologies capable of removing both organic micropollutants and antibiotic-resistant bacteria.

## Data Availability

The data that support the findings of this study are available from the corresponding author upon reasonable request.

## Supporting Information Available

Detailed information about hydroponic solutions; physicochemical properties of pharmaceuticals; extraction and LC-MS/MS methods and MRM transitions; heatmaps of pharmaceutical distribution in lettuce samples; statistical and ecotoxicological results; health risk estimation, and antimicrobial resistance risk quotients (PDF)
