# Supplementary material for: Assessing Lettuce Exposure to a Multipharmaceutical Mixture under Hydroponic Conditions: Findings through LC-ESI-TQ Analysis and Ecotoxicological Assessments
Source: ACS Omega. 2024 Nov 29;9(50):49707–18. doi: 10.1021/acsomega.4c08013 (PMC11656385; doi:10.1021/acsomega.4c08013)
Supplement: Supplementary file 1 — ao4c08013_si_001.pdf [file ao4c08013_si_001.pdf]

Supporting Information for

Assessing Lettuce Exposure to a Multi-Pharmaceutical Mixture under Hydroponic Conditions: Findings through LC-ESI-TQ Analysis and Ecotoxicological Assessments

Ludmila Mravcová[1], Vojtěch Jašek[2], Marie Hamplová[1], Jitka Navrkalová[1], Anna Amrichová[1], Helena Zlámalová Gargošová[1], Jan Fučík[1,*]

[1] *Institute of Chemistry and Technology of Environmental Protection, Faculty of Chemistry, Brno University of Technology, Purkyňova 118, 612 00 Brno, Czech Republic*

[2] *Institute of Materials Chemistry, Faculty of Chemistry, Brno University of Technology, Purkyňova 118, 612 00 Brno, Czech Republic*

*corresponding author: *xcfucikj@vutbr.cz*

Table of Contents

Table S1. Modified Sonneveld's recipe for the hydroponic solution (tank A)

Table S2. Modified Sonneveld's recipe for the hydroponic solution (tank B)

Table S3. Physico-chemical properties of pharmaceuticals

Figure S1-S4. *L. Sativa images* during its growth in hydroponic solutions

Appendix 1. Extraction Methods

Appendix 2. LC-MS/MS method

Table S4. MRM transitions of selected pharmaceuticals for LC-MS/MS analysis

Figure S5-S9. Heatmap of pharmaceutical distribution in lettuce samples over a 35-day period at various concentrations

Figure S10. Principal component analysis of pharmaceutical residues in *L. sativa* roots and leaves across different exposure times

Table S5. Comparison of BCFs and TFs at various sampling days using T-test

Figure S11. Length of Lettuce Roots at different pharmaceutical concentrations at sampling days 14; 21; 28 and 35

Figure S12. Photo A and B of *L. Sativa* Root Morphology at Different Concentrations

Figure S13. PLS-DA of ecotoxicological endpoints for *L. sativa* grown under hydroponic conditions exposed to various concentrations of pharmaceutical mixture

Appendix 3. Health Risk Assessment - Monte Carlo Simulation

Table S6. Input Parameters for Mone Carlo Simulation

Table S7. Health Risk Assessment - Monte Carlo Simulation

Figure S14. Cumulative distribution of Risk Quotients (RQs) and Hazard Indexes (HIs) of pharmaceuticals at a water concentration of 10 $\mu g \cdot L^{-1}$ for adults.

Figure S15. Cumulative distribution of Risk Quotients (RQs) and Hazard Indexes (HIs) of pharmaceuticals at a water concentration of 50 $\mu g \cdot L^{-1}$ for adults.

Appendix 4. Calculation Procedure to Estimate Potential Risk towards Antimicrobial Resistance

Table S8. Risk Quotients towards Emergence of Antimicrobial Resistance

This document contains in total: 24 pages, 8 Tables, 15 Figures

Modified Sonneveld's recipe for the hydroponic solutions

Note: Tank A and Tank B salts are mixed in ratio 1:1 to EC value as stated in Main Manuscript of the Article, subsection 2.2.

Table S1. Modified Sonneveld's recipe for the hydroponic solution (tank A, Total Volume 2.5 L) [1]

Salt	Weight [g]
$Ca(NO_3)_2 \cdot 3H_2O$	121.70
NH_4NO_3	9.50
KNO_3	110.6
$FeSO_4 \cdot 7H_2O$	0.16
diethylenetriaminepentaacetic acid	0.22

Table S2. Modified Sonneveld's recipe for the hydroponic solution (tank B, Total Volume 2.5 L) [1]

Salt	Weight [g]
KH_2PO_4	34.10
$MgSO_4 \cdot 7H_2O$	61.60
$MnSO_4 \cdot H_2O$	0.19
H_3BO_3	0.23
$Na_2MoO_4 \cdot 2H_2O$	0.02
$ZnSO_4 \cdot 7H_2O$	0.14
$CuSO_4 \cdot 5H_2O$	0.02

Table S3. Physico-chemical properties of pharmaceuticals [2,3]

Pharmaceutical group	Substance name	Chemical formula	Mw [-]	pKa	log P	Solubility in water [mg·L^{-1}]
Beta blockers	acebutolol	$C_{18}H_{28}N_2O_4$	336.4	9.5	1.7	259
	atenolol	$C_{14}H_{22}N_2O_3$	266.3	9.6	0.2	40
	nadolol	$C_{17}H_{27}NO_4$	309.4	9.7	0.7	46.4
	propranolol	$C_{16}H_{21}NO_2$	259.3	9.5	3.0	61.7
Fluoroquinolone antibacterials	ciprofloxacin	$C_{17}H_{18}FN_3O_3$	331.3	6.1; 8.7	-1.1	<1
	enrofloxacin	$C_{19}H_{22}FN_3O_3$	359.4	5.55; 7.24	-0.2	53.9
	moxifloxacin	$C_{21}H_{24}FN_3O_4$	401.4	6.3; 9.1	0.6	1,146
	norfloxacin	$C_{16}H_{18}FN_3O_3$	319.3	6.1; 8.8	-1.0	250,000
	ofloxacin	$C_{18}H_{20}FN_3O_4$	361.4	6.0; 9.3	-0.4	28,300
	pefloxacin	$C_{17}H_{20}FN_3O_3$	333.4	5.55; 7.01	0.3	11,400
Macrolides	azithromycin	$C_{38}H_{72}N_2O_{12}$	749.0	8.5	4.0	2.37
	clarithromycin	$C_{38}H_{69}NO_{13}$	748.0	9.0	3.2	0.33
	roxithromycin	$C_{41}H_{76}N_2O_{15}$	837.0	9.3	1.7	0.0189
Nonsteroidal anti-inflammatory drugs	naproxen	$C_{14}H_{14}O_3$	230.3	4.2	3.3	15.9
Sulfonamides and Trimethoprim	sulfacetamide	$C_8H_{10}N_2O_3S$	214.2	2.14; 4.3	-1.0	32.1
	sulfadimethoxine	$C_{12}H_{14}N_4O_4S$	310.3	1.99; 6.91	1.6	343
	sulfamethoxazole	$C_{10}H_{11}N_3O_3S$	253.3	1.6; 5.7	0.9	610
	sulfapyridine	$C_{11}H_{11}N_3O_2S$	249.3	8.4	0	33.1
	trimethoprim	$C_{14}H_{18}N_4O_3$	290.3	7.1	0.9	400
Tetracyclines	tetracycline	$C_{22}H_{24}N_2O_8$	444.4	3.3; 7.7	-2.0	231

Lettuce Images During Growth

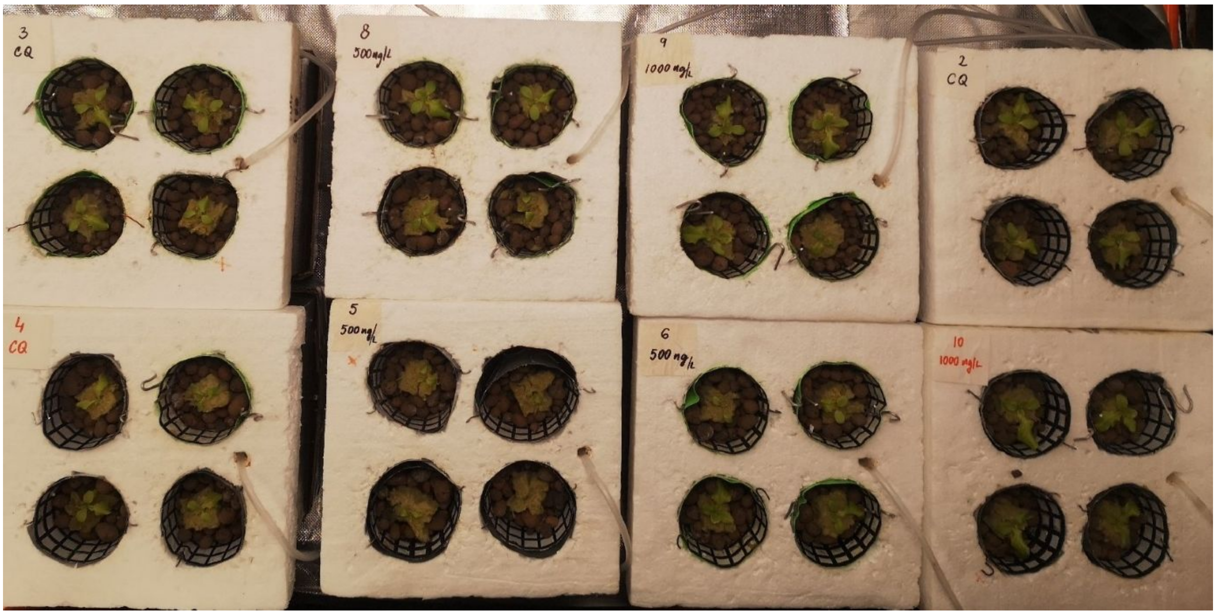

Figure S1. *L. Sativa* plants after 7 days of growth in a hydroponic solution

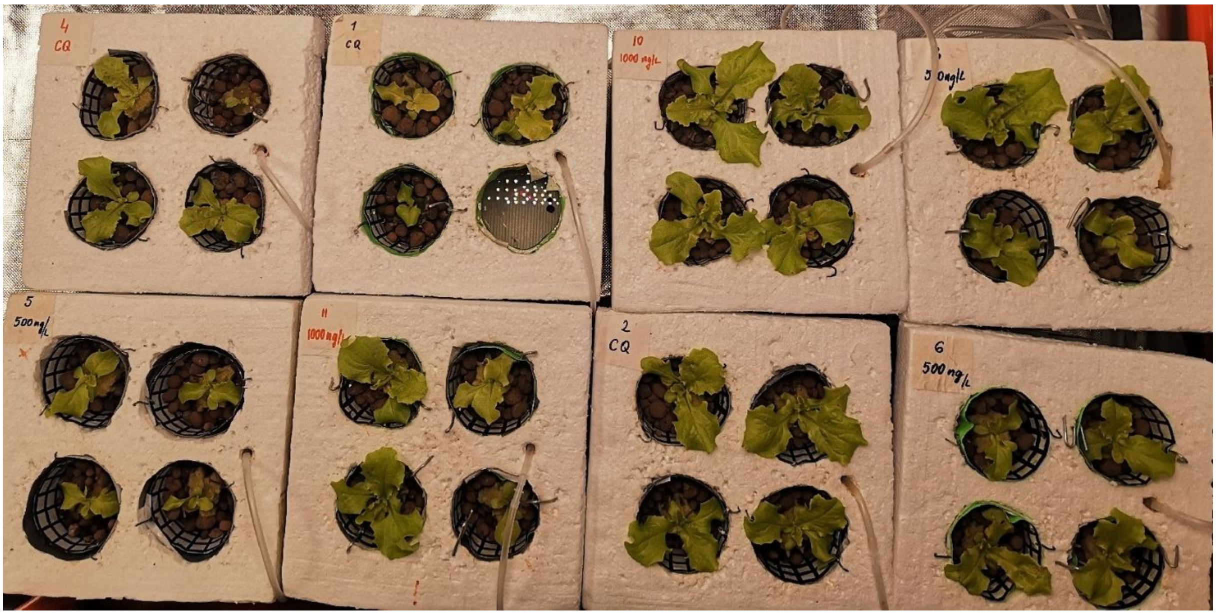

Figure S2. *L. Sativa* plants after 14 days of growth in a hydroponic solution

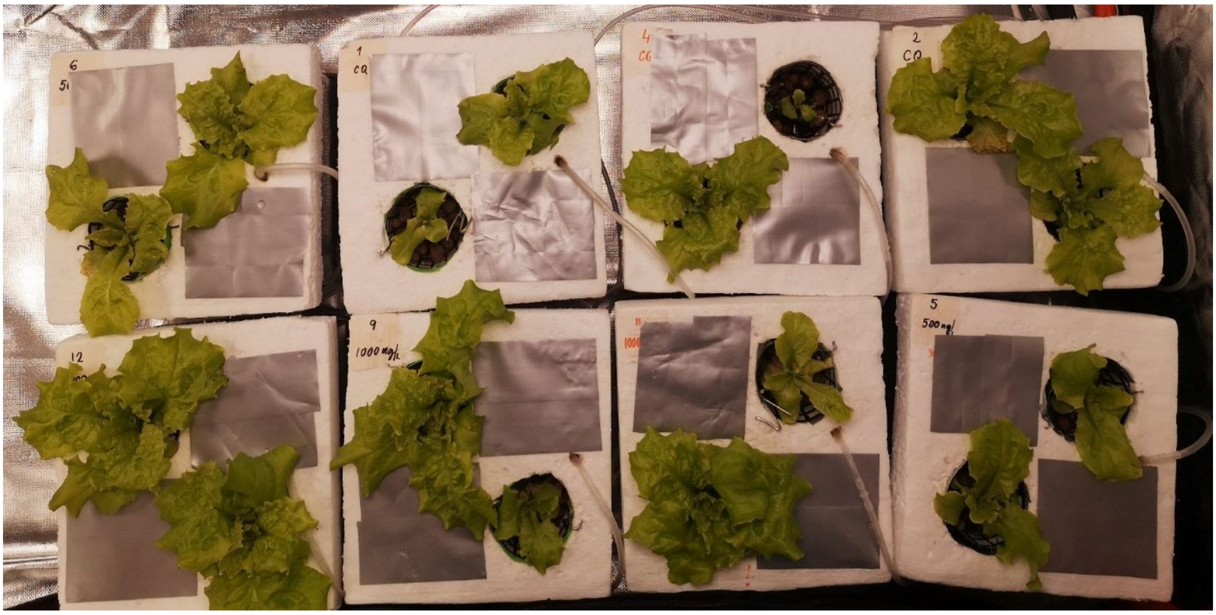

Figure S3. *L. Sativa* plants after 28 days of growth in a hydroponic solution

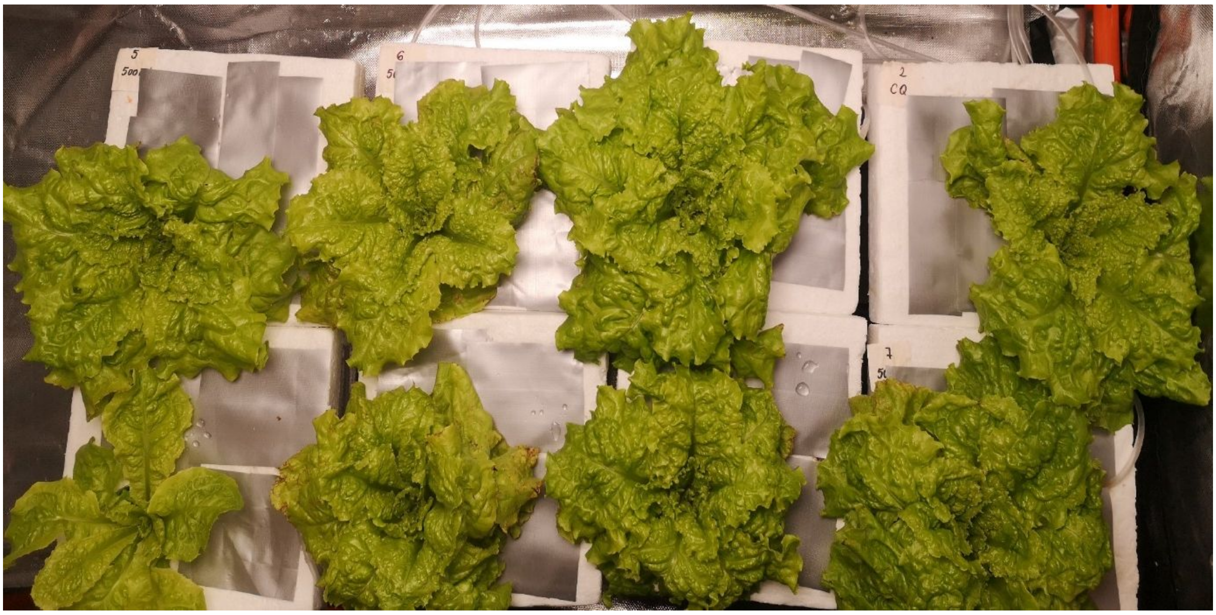

Figure S4. *L. Sativa* plants after 35 days of growth in a hydroponic solution

Appendix 1. Extraction Methods

QuEChERS Extraction of Lettuce Samples

The lettuce leaves and roots were extracted separately using already validated and published method. [4] Briefly, 0.1 g of lyophilized and homogenized lettuce leaves were accurately weighed and placed into a 50 mL PE centrifugation tube. In the **extraction step**, ceramic homogenizers were initially introduced, followed by pipetting of 5 mL of the extraction medium (MeOH:McIlvaine buffer, pH 2.6:ACN in a ratio of 8:20:72). Following this, the sample was vortexed for 1 min. Subsequently, separation salts (2 g anhydrous Na_2SO_4 and 0.5 g NaCl) were added, and the mixture was further vortexed for 1.5 min before centrifugation at 3,500 rpm for 10 min at 20 °C. In the **purification step**, following centrifugation, 2 mL of the organic phase was carefully pipetted into a 15 mL PE centrifugation tube preloaded with dSPE sorbents (12.5 mg DSC-18, 12.5 mg PSA, and 225 mg of anhydrous Na_2SO_4). Subsequently, the sample was vortexed for 1 min, followed by another centrifugation at 3,500 rpm for 10 min at 20 °C. Finally, the sample was filtered through 0.22 μm nylon syringe filters (diameter 13 mm) into a 2-mL glass vial, ready for LC-MS/MS analysis.

Appendix 2. LC-MS/MS method

Lettuce extracts were analyzed using a previously validated and published method for over 40 pharmaceuticals.[4] However, in this study, only 20 pharmaceuticals were analyzed, which allowed for lower limits of detection (LoDs) and quantification (LoQs) to be achieved, while maintaining similar recovery rates, as the extraction method remained the same.

Instrumental analysis for the quantification of PhACs in lettuce and soil extracts was performed using ultra-performance liquid chromatography (UHPLC Agilent 1290 Infinity LC) coupled with a triple quadrupole mass spectrometer (Bruker EVOQ LC-TQ) with electrospray ionization (ESI). The gas sources of nitrogen and air were provided by an external gas generator (Peak Scientific – Genius 3045).

Chromatographic separation was accomplished using a Luna® Omega Polar C18 Phenomenex column (100 x 2.1 mm, 1.6 μm). The column temperature was optimized at 35°C, and the flow rate was set to 0.5 $mL \cdot min^{-1}$. The mobile phases consisted of A) 0.1% FA in H_2O and B) ACN, following a gradient program for the A eluent (%): t(0 min) = 90, t(0.5 min) = 90, t(13.0 min) = 35, t(14.0 min) = 10, and t(15.5 min) = 90. The LC method was set to a stop time of 16 min, with a 2-min re-equilibration time. The injection volume for all analyses was 7 μL. To prevent carry-over, an external needle wash was performed using a wash solvent composed of FA:H_2O:ACN at a ratio of 1:9:90 for 30 s.

The MS conditions were set as follows for electrospray ionization in positive mode: spray voltage: 4,500 V; cone temperature: 350°C; cone gas flow: 15 arbitrary units (a.u.); heated probe temperature: 500°C; probe gas flow: 25 a.u.; nebulizer gas flow: 45 a.u.; and exhaust gas: ON. For both quantitative and qualitative analysis of PhACs, the multiple reaction monitoring (MRM) mode was employed, using the specific MRM transitions outlined in Table S4. Argon served as the collision gas at a pressure of 1.5 mTorr.

Table S4. MRM transitions of selected pharmaceuticals for LC-MS/MS analysis

**Analyte Name**	**RT [min]**	**Quantitative transition**			**Quantitative transition**		
		**Precursor ion [m/z]**	**Productn ion [m/z]**	**CE [eV]**	**Precursor ion [m/z]**	**Product ion [m/z]**	**CE [eV]**
acebutolol	3.65	337.2	116.0	20.0	337.2	319.0	10.0
atenolol	1.17	267.0	145.2	20.0	267.0	190.1	10.0
nadolol	2.50	310.2	253.8	10.0	310.2	200.8	20.0
propranolol	5.42	259.3	116.0	15.0	259.3	183.0	15.0
ciprofloxacin	3.35	332.4	314.2	10.0	332.4	288.2	10.0
enrofloxacin	3.78	360.4	316.2	10.0	360.4	342.2	20.0
moxifloxacin	4.83	402.0	358.2	20.0	402.0	384.2	20.0
norfloxacin	3.22	320.1	276.1	10.0	320.1	231.1	40.0
ofloxacin	3.25	362.2	318.1	10.0	362.2	261.1	20.0
pefloxacin	3.34	334.0	317.0	10.0	334.0	289.0	10.0
azithromycin	4.84	375.2	591.3	10.0	375.2	158.0	20.0
clarithromycin	7.54	748.5	158.1	20.0	748.5	590.4	10.0
roxithromycin	8.03	837.5	679.5	20.0	837.5	157.9	30.0
naproxen	8.39	231.0	185.2	10.0	231.0	169.0	20.0
sulfacetamide	1.62	215.2	156.0	5.0	215.2	108.2	10.0
sulfadimethoxine	5.48	255.0	156.0	10.0	255.0	92.1	15.0
sulfamethoxazole	4.14	254.3	156.0	10.0	254.3	108.1	20.0
sulfapyridine	1.97	250.3	156.0	10.0	250.3	92.2	20.0
trimethoprim	2.68	291.2	230.1	20.0	291.2	261.0	20.0
tetracycline	5.00	445.0	427.2	10.0	445.0	428.0	10.0

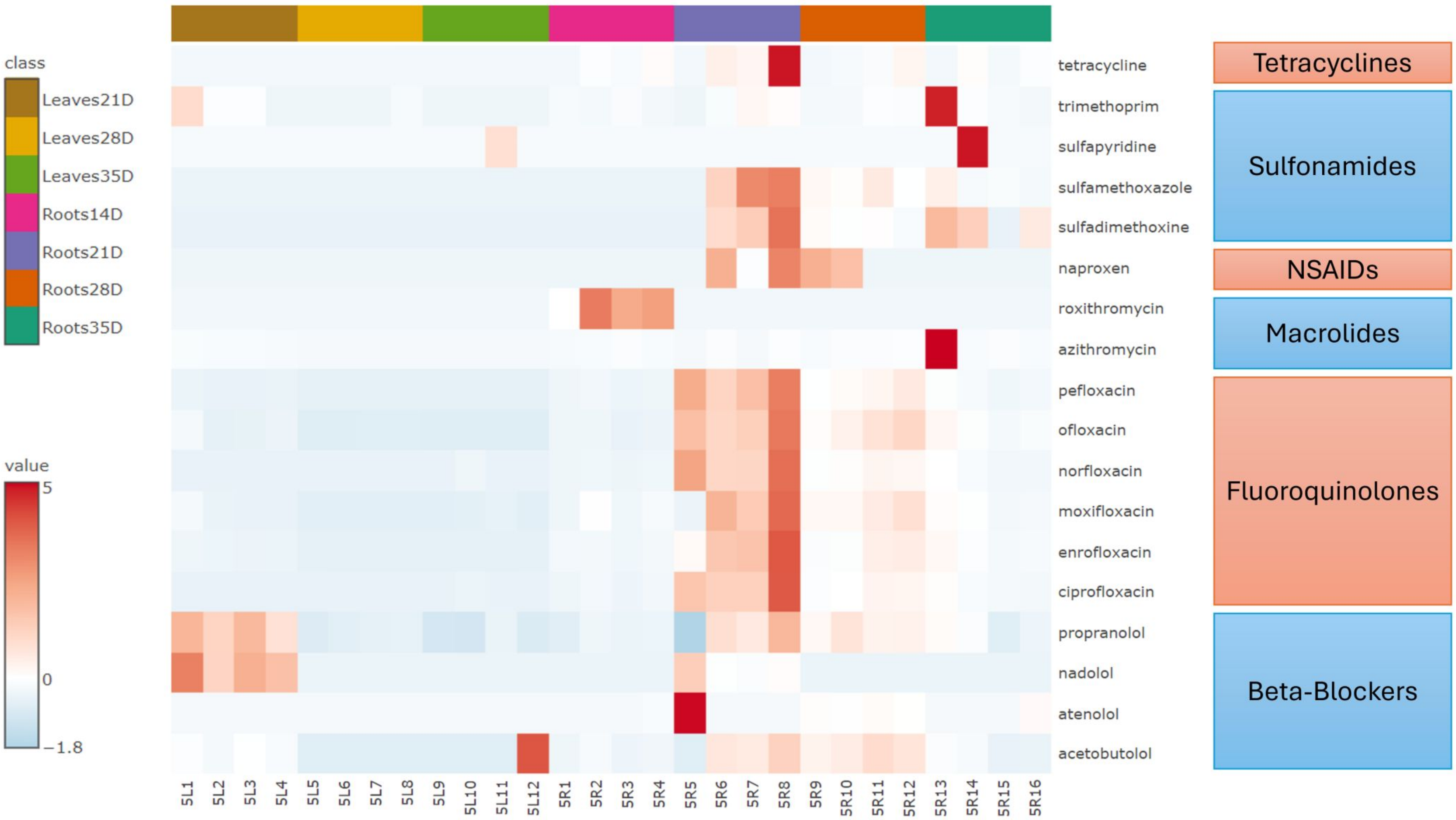

Figure S5. Heatmap of pharmaceutical distribution in lettuce samples over a 35-day period at concentration of 5 µg·L^{-1}, created using Metabo Analyst[5]

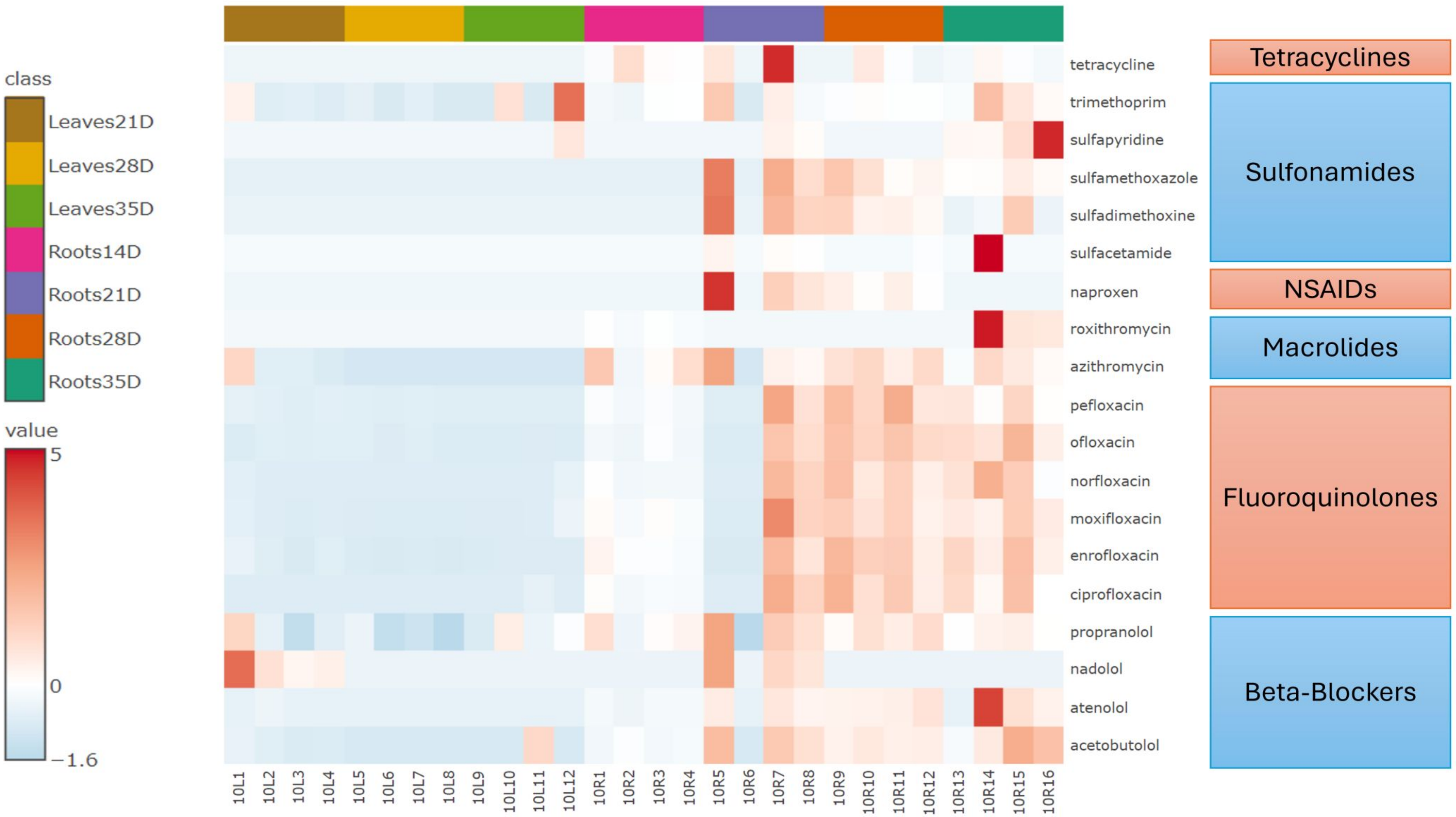

Figure S6. Heatmap of pharmaceutical distribution in lettuce samples over a 35-day period at concentration of 10 µg·L^{-1}, created using Metabo Analyst[5]

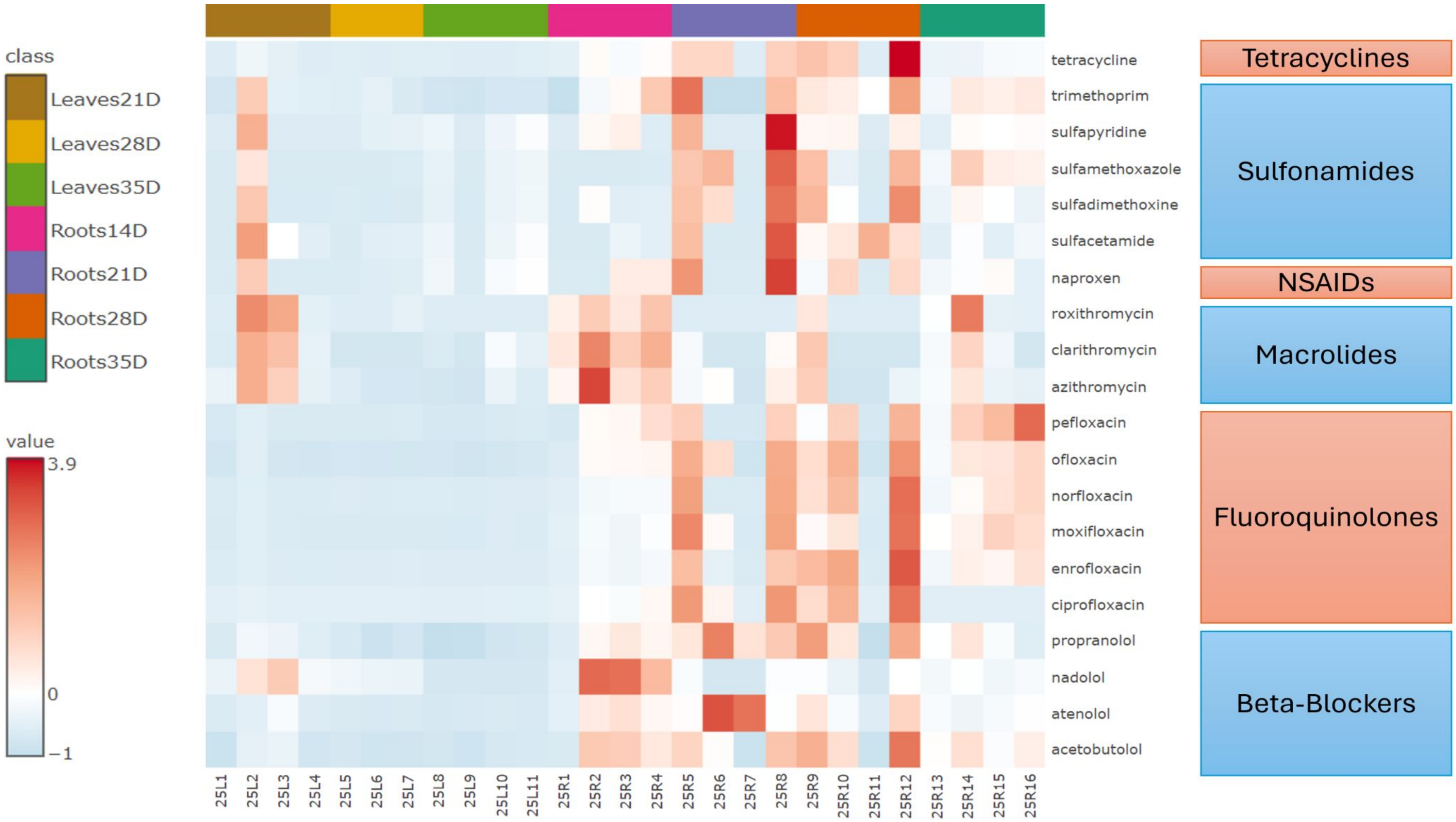

Figure S7. Heatmap of pharmaceutical distribution in lettuce samples over a 35-day period at concentration of 25 μg·L^{-1}, created using Metabo Analyst[5]

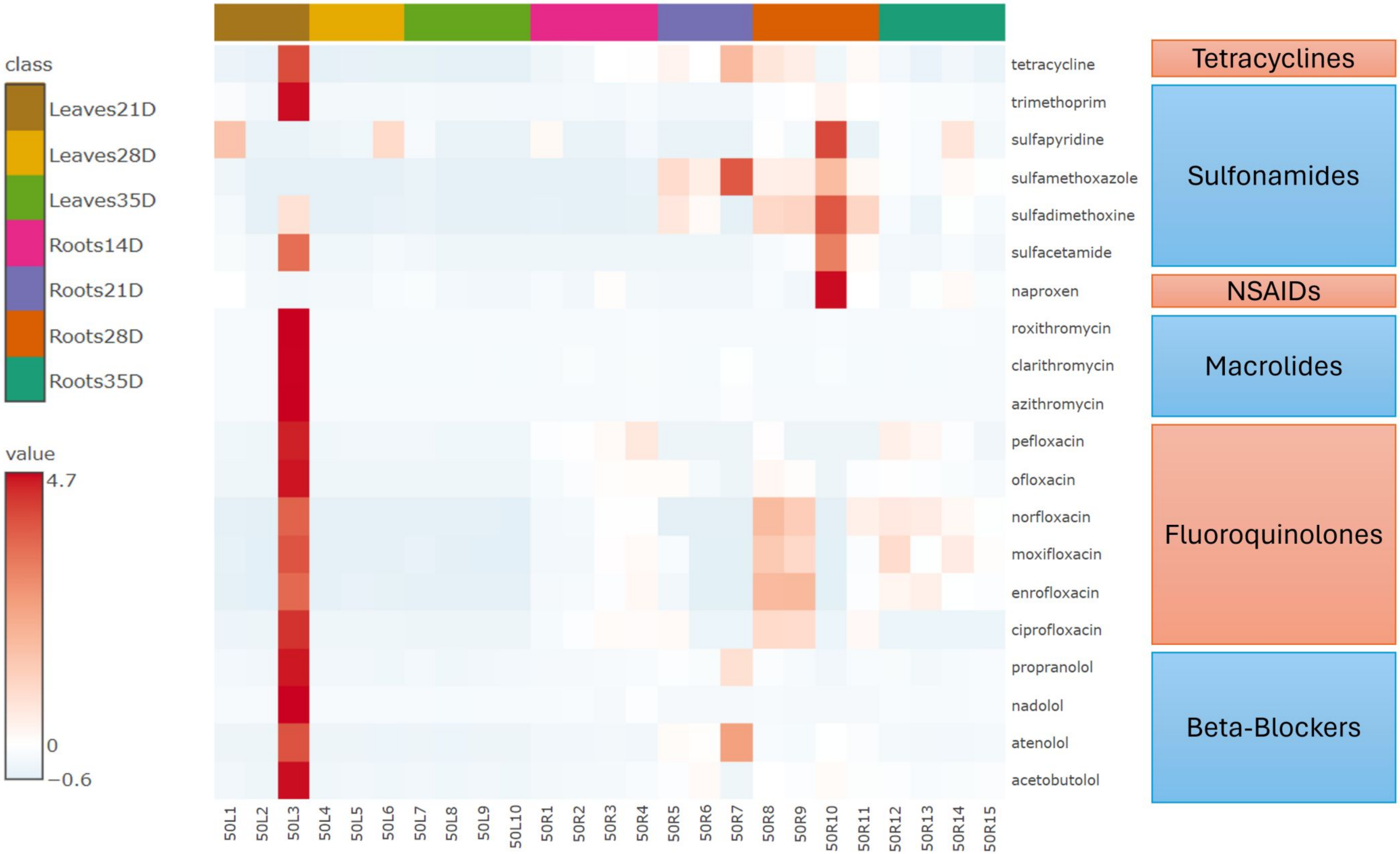

Figure S8. Heatmap of pharmaceutical distribution in lettuce samples over a 35-day period at concentration of 50 $\mu g \cdot L^{-1}$, created using Metabo Analyst[5]

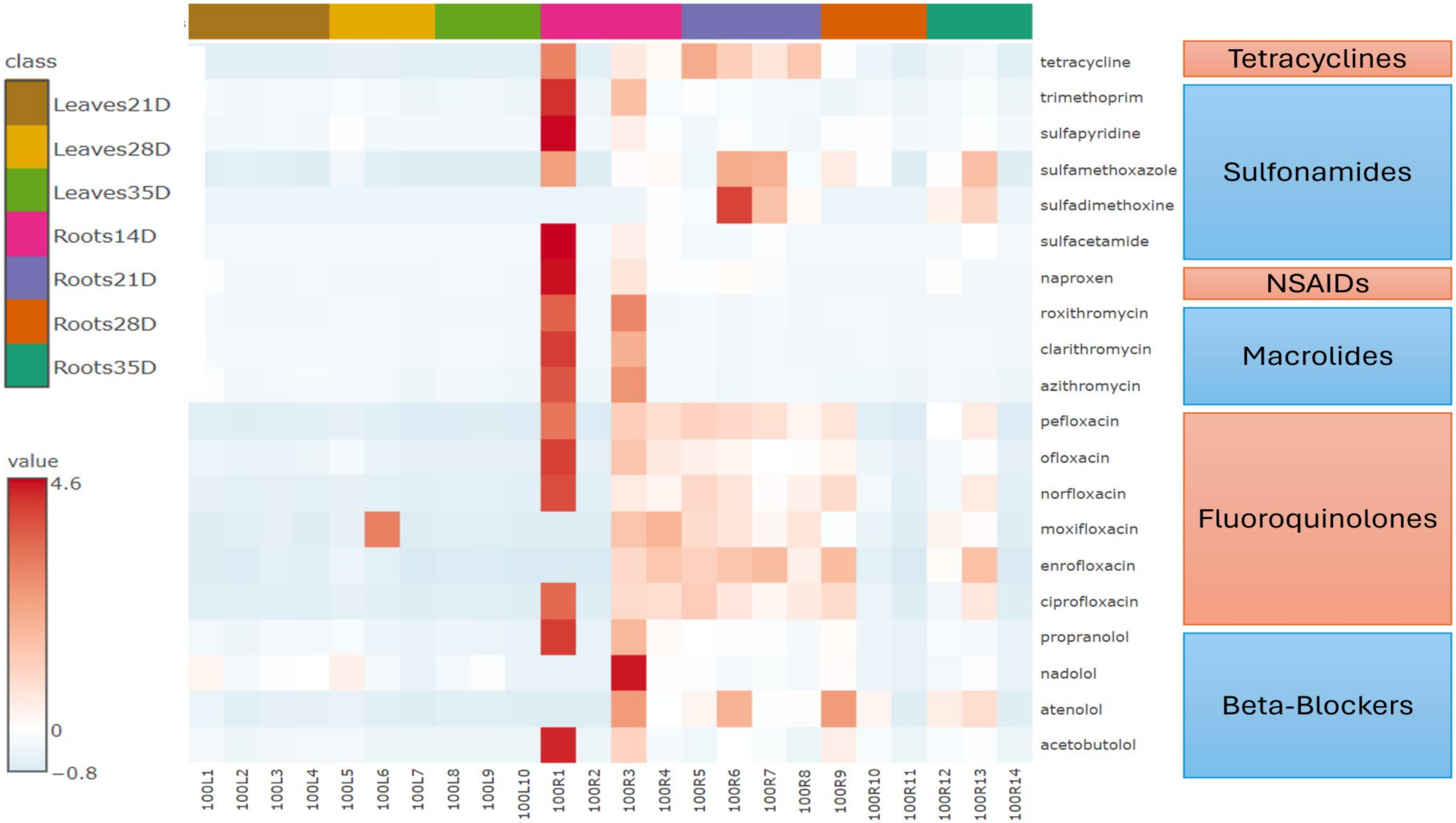

Figure S9. Heatmap of pharmaceutical distribution in lettuce samples over a 35-day period at concentration of 100 $\mu g \cdot L^{-1}$, created using Metabo Analyst[5]

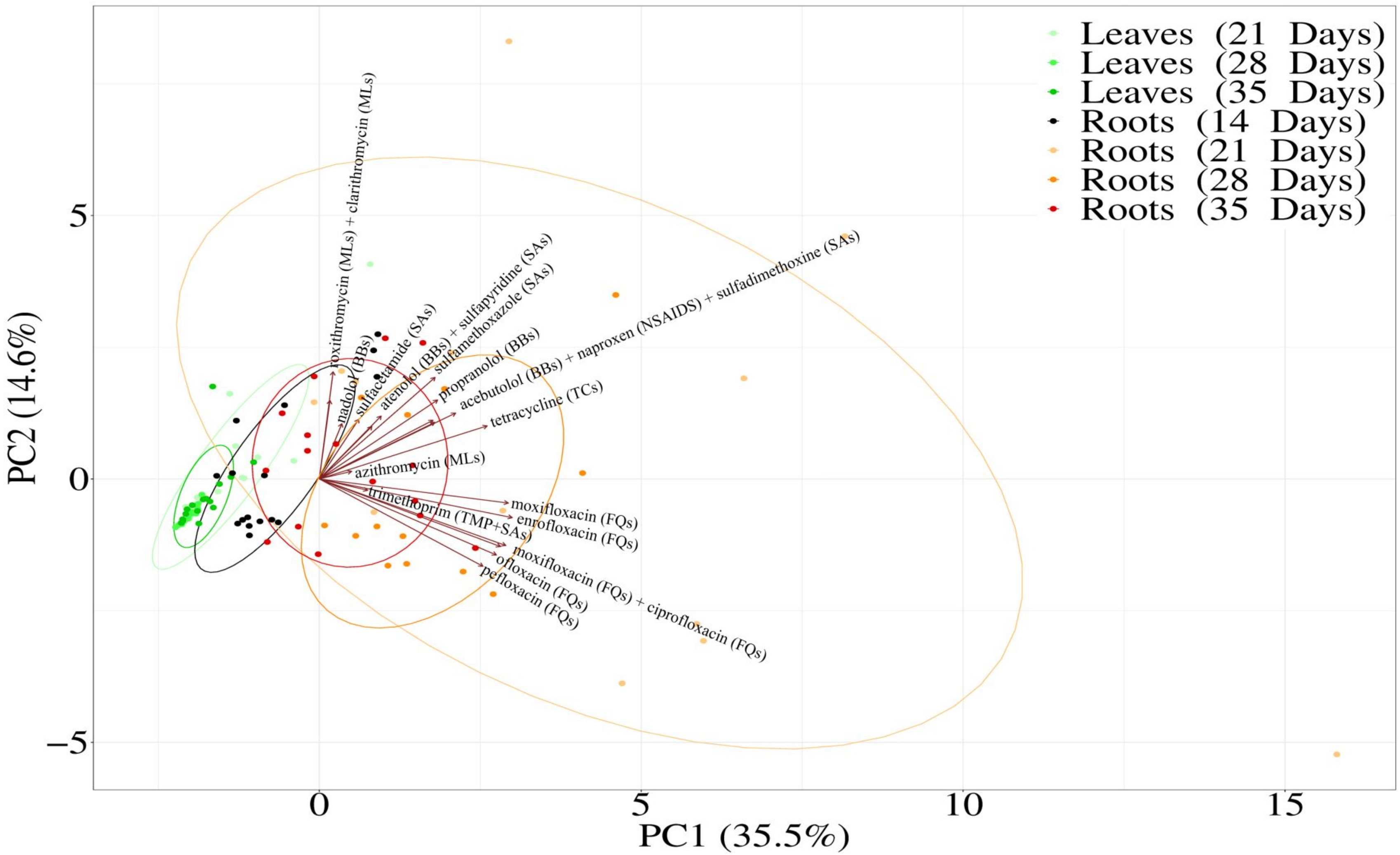

Figure S10. Principal Component Analysis of pharmaceutical residues in *L. sativa* roots and leaves across different exposure times, created using SRplot[6]

Table S5. Comparison of BCFs and TFs at various sampling days using T-test

Pharmaceutical Name	BCF Comparison						TF Comparison		
	14D vs 21D	14D vs 28D	14D vs 35D	21D s 28D	21D vs 35D	28D vs 35D	21D vs 28D	21D vs 35D	28D vs 35D
Acetobutolol	TRUE	TRUE	TRUE	FALSE	TRUE	TRUE	FALSE	TRUE	FALSE
Atenolol	TRUE	TRUE	TRUE	TRUE	TRUE	TRUE	TRUE	TRUE	FALSE
Azithromycin	TRUE	TRUE	TRUE	TRUE	TRUE	FALSE	N.D.	TRUE	N.D.
Ciprofloxacin	TRUE	TRUE	TRUE	TRUE	TRUE	FALSE	FALSE	TRUE	FALSE
Clarithromycin	N.D.	FALSE	TRUE	N.D.	N.D.	TRUE	N.D.	N.D.	N.D.
Enrofloxacin	TRUE	TRUE	TRUE	TRUE	TRUE	FALSE	FALSE	FALSE	FALSE
Moxifloxacin	TRUE	TRUE	FALSE	TRUE	TRUE	TRUE	FALSE	TRUE	TRUE
Nadolol	TRUE	TRUE	TRUE	FALSE	FALSE	FALSE	TRUE	FALSE	N.D.
Naproxen	TRUE	TRUE	TRUE	FALSE	TRUE	TRUE	TRUE	FALSE	N.D.
Norfloxacin	TRUE	TRUE	TRUE	TRUE	TRUE	TRUE	FALSE	FALSE	FALSE
Ofloxacin	TRUE	TRUE	TRUE	FALSE	FALSE	TRUE	TRUE	FALSE	TRUE
Pefloxacin	N.D.	TRUE	TRUE	N.D.	N.D.	FALSE	FALSE	FALSE	FALSE
Propranolol	TRUE	FALSE	FALSE	TRUE	TRUE	FALSE	TRUE	FALSE	TRUE
Roxithromycin	N.D.	FALSE	FALSE	N.D.	N.D.	FALSE	N.D.	N.D.	N.D.
Sulfacetamide	N.D.	N.D.	N.D.	N.D.	N.D.	TRUE	N.D.	N.D.	N.D.
Sulfadimethoxine	TRUE	TRUE	TRUE	TRUE	TRUE	TRUE	N.D.	N.D.	N.D.
Sulfamethoxazole	TRUE	TRUE	TRUE	TRUE	TRUE	TRUE	N.D.	N.D.	N.D.
Sulfapyridine	N.D.	TRUE	TRUE	N.D.	N.D.	FALSE	N.D.	N.D.	N.D.
Tetracycline	TRUE	TRUE	FALSE	TRUE	TRUE	TRUE	N.D.	FALSE	N.D.
Trimethoprim	TRUE	TRUE	FALSE	TRUE	TRUE	TRUE	FALSE	FALSE	FALSE

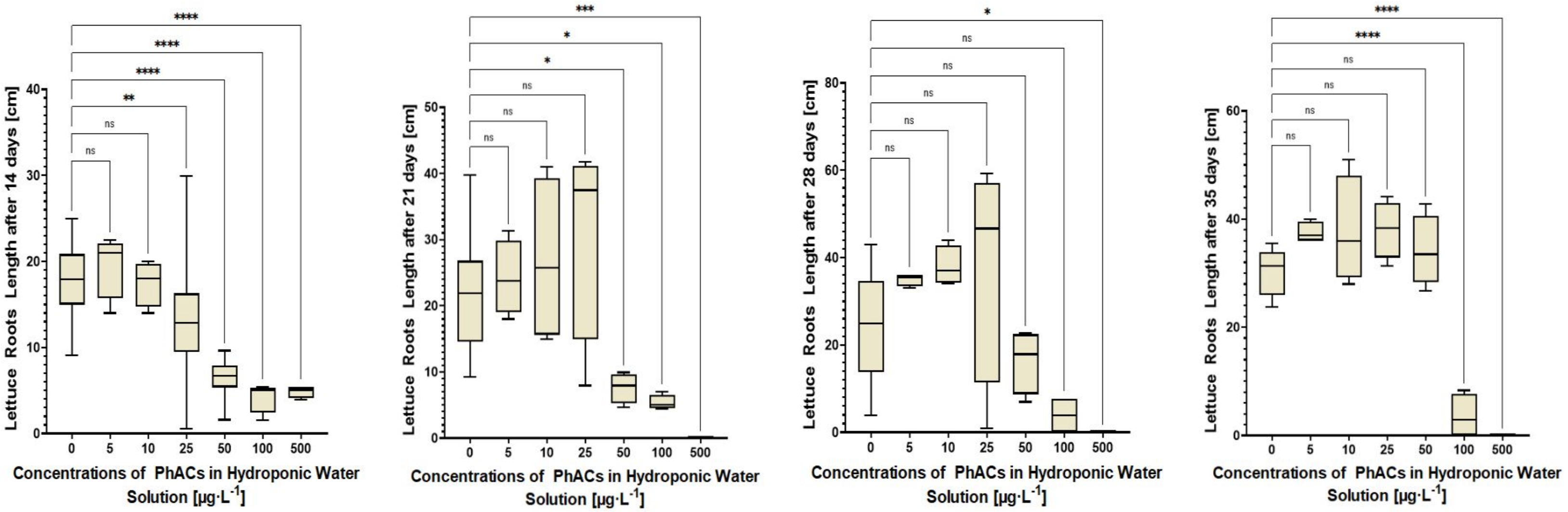

Figure S11. Length of Lettuce Roots at different pharmaceutical concentrations at sampling days 14; 21; 28 and 35

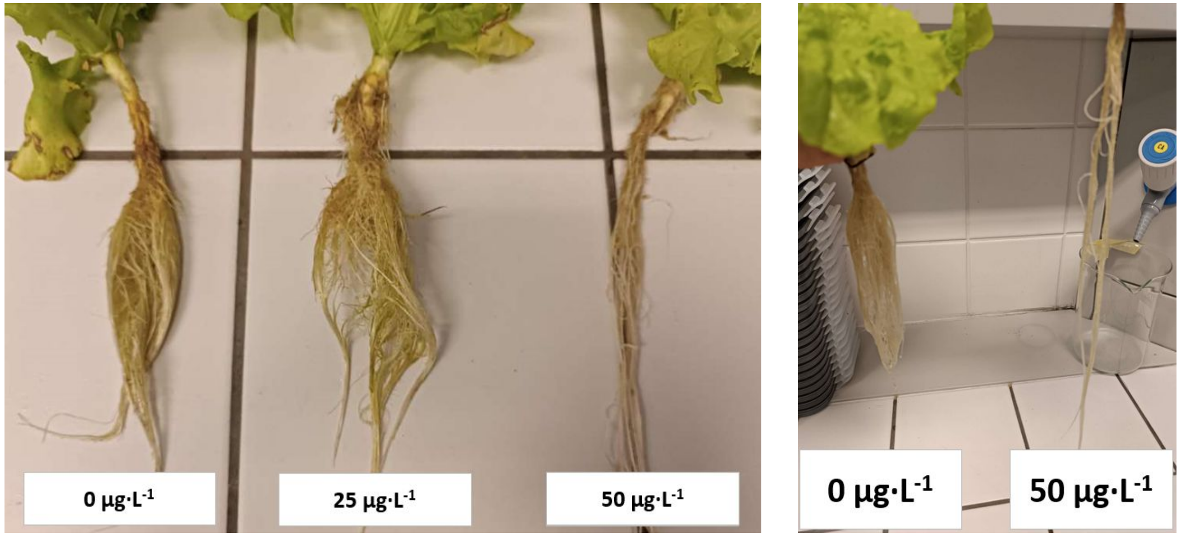

Figure S12. Photo A and B of *L. Sativa* Root Morphology at Different Concentrations

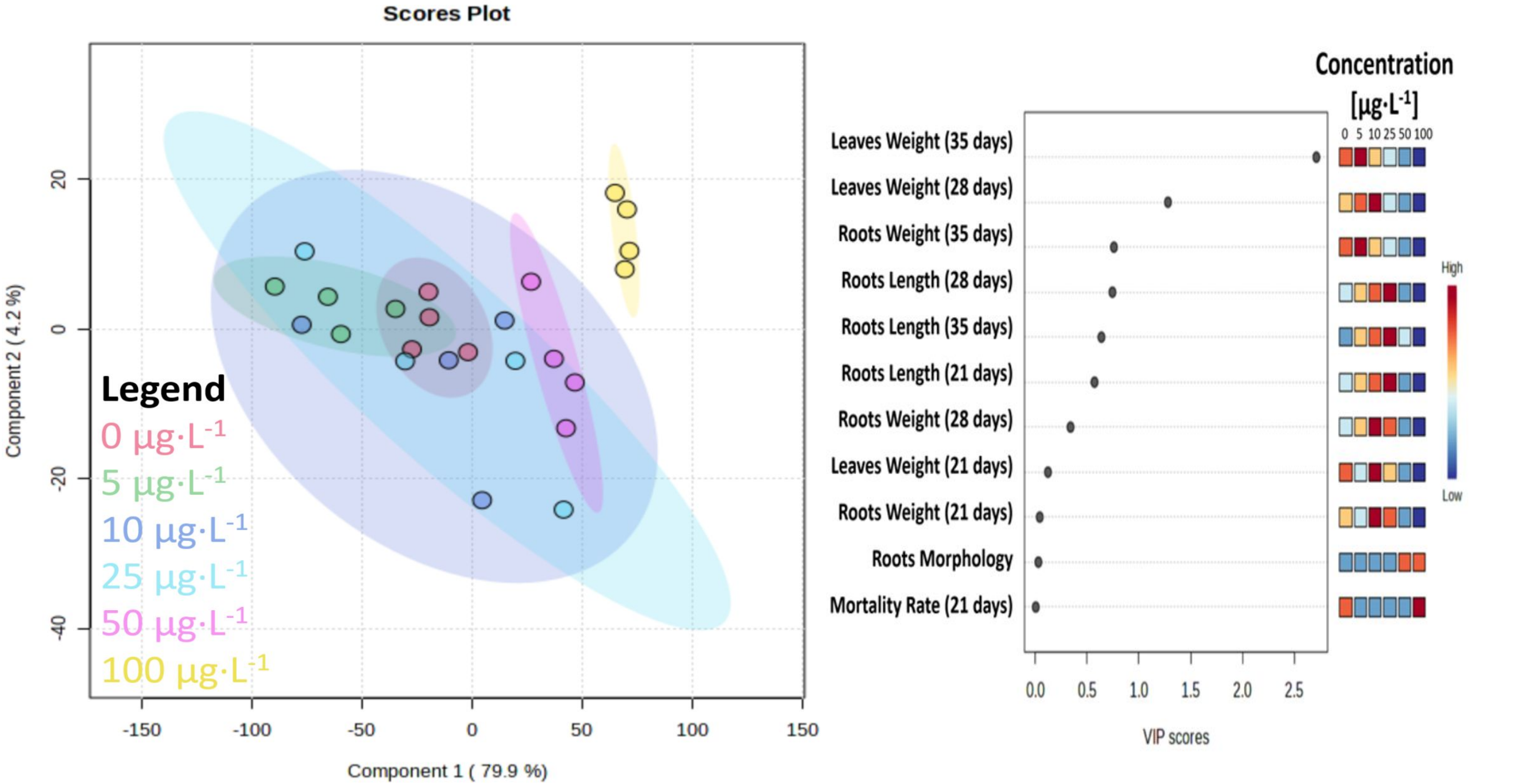

Figure S13. Partial least squares – discriminant analysis (PLS-DA) of ecotoxicological endpoints for *L. sativa* grown under hydroponic conditions exposed to various concentrations of pharmaceutical mixture, created using Metabo Analyst[5]

Appendix 3. Health Risk Assessment - Monte Carlo Simulation

Risk Quotient and Hazard Index Calculation

Firstly, the estimated daily intake (EDI) for each pharmaceutical was calculated using time-weighted average water concentrations, as detailed in the Main Manuscript, subsection 3.1. We derived the EDI for hydroponically grown lettuces at two concentration levels, specifically at 10 and 50 $\mu g \cdot L^{-1}$ after 35 days. To estimate pharmaceutical concentrations in lettuce leaves, we used Eq. 1, taking into account initial concentrations and pharmaceutical degradation rates, and applied the calculated bioaccumulation factors (BCFs) and translocation factors (TFs) from the main manuscript, as detailed in the Main Manuscript, subsection 3.1.

Consequently, the concentration of pharmaceuticals in lettuce leaves was then used in Eq. 2, altogether with the average water content in lettuce leaves of 95.3±0.6%. [7–9] The average daily intake of lettuce per unit body weight per day is 0.25±0.01 $ng \cdot kg^{-1} \cdot day^{-1}$, as reported in study.[9]

$$C_{\text{lettuce leaves}} = \text{BCF} \cdot \text{TF} \cdot C_{water\ avg.} \qquad (1)$$

where $C_{\text{lettuce leaves}}$ represents the concentration of pharmaceutical in lettuce leaves ($ng \cdot g^{-1}$ dry weight). BCF stands for bioconcentration factor ($L \cdot kg^{-1}$), indicating the ratio of the pharmaceutical concentration in lettuce roots to the water. TF represents the translocation factor (-), measuring the extent to which pharmaceuticals are transferred from lettuce roots to leaves. $C_{\text{water avg.}}$ denotes the time-weighted average concentration of the pharmaceutical in the water ($\mu g \cdot L^{-1}$).

$$\text{EDI} = C_{\text{lettuce leaves}} \cdot \beta_{fw/dw} \cdot IR_{lettuce} \qquad (2)$$

where EDI stands for estimated daily intake ($ng \cdot kg^{-1} \cdot day^{-1}$), $C_{\text{lettuce leaves}}$ represents the concentration of pharmaceutical in lettuce leaves ($ng \cdot g^{-1}$ dry weight), $\beta_{fw/dw}$ denotes the conversion factor from dry weight to fresh weight (-) and $IR_{lettuce}$ is the average daily intake of lettuce ($g \cdot kg^{-1} \cdot day^{-1}$).

Consequently, we searched scientific literature for acceptable daily intake (ADI) values (Table S7) [10–14] to establish benchmarks for calculating risk quotients (RQs). Using these values, we calculated the RQ for each pharmaceutical separately with Eq. 3. Next, we computed the Hazard Index (HI) as the sum of all RQs for each water contamination level using Eq. 4 (Table S7), as described in studies [7,15]. A human health risk is considered negligible when the RQ or HI is less than 0.01, considerable when either RQ or HI exceeds 0.01, and distinct when either value is greater than 0.05.[15]

$$\mathrm{RQ} = \frac{EDI}{ADI} \tag{3}$$

where RQ is risk quotient, EDI is estimated daily intake and ADI stands for acceptable daily intake.

$$HI = \sum RQ \tag{4}$$

where HI stands for hazard index and RQ denotes risk quotient.

Monte Carlo Simulation

A Monte Carlo simulation is a valuable computational method that uses statistical sampling techniques to estimate a probabilistic solution to a mathematical model (Eqs 1-4 and Table S6).[16,17] In our case, this approach was used to estimate the risk of pharmaceutical residue intake through food, assessing potential health risks to humans (Table S7, Figures S14-S15). In this study, the Monte Carlo simulation analysis was done in 100,000 iterations by Minitab Workspace.

Table S6. Input Parameters for Mone Carlo Simulation

Parameter	Unit	Description	Distribution	Values	Reference
BCF	$[L \cdot kg^{-1}]$	Bioaccumulation factor	Normal	Varied by pharmaceutical, as determined in this manuscript (data in Table 1)	This manuscript
TF	[-]	Translocation factor	Normal		This manuscript
$C_{water,\ avg.}$	$[\mu g \cdot L^{-1}]$	The time-weighted average concentration of the pharmaceutical in the water	Normal		This manuscript
$\beta_{fw/dw}$	[-]	The conversion factor from dry weight to fresh weight of lettuce	Normal	95.3±0.6	7–9
$IR_{lettuce}$	$[g \cdot kg^{-1} \cdot day^{-1}]$	Average daily intake of lettuce per unit body weight per day	Lognormal	0.25±0.01	9
ADI	$[ng \cdot kg^{-1} \cdot day^{-1}]$	Acceptable daily intake of hparmaceutical residues	Fixed	Varied by pharmaceutical, (data in Table S7)	10–14

Table S7. Health Risk Assessment - Monte Carlo Simulation - Acceptable daily intake (ADI) for selected pharmaceuticals, estimated risk quotients (RQ) for each compound and total sum of RQs as hazard risk (HI) at different concentrations of water contamination, N.D. – not determined.

Pharmaceutical Group	Pharmaceutical Name	ADI $[ng \cdot kg^{-1} \cdot day^{-1}]$	RQ mean value At 10 $\mu g \cdot L^{-1}$ for adult [-]	RQ mean value at 50 $\mu g \cdot L^{-1}$ for adult [-]
Beta-Blockers	acetobutolol	2900 [10]	0.000194	0.000974
	atenolol	400 [11]	0.000886	0.004449
	nadolol	500 [10]	N.D.	N.D.
	propranolol	430 [10]	0.005103	0.25508
Fluoroquinolones	ciprofloxacin	1600 [12]	0.001576	0.007927
	enrofloxacin	2000 [13]	0.001993	0.009974
	moxifloxacin	5714 [14]	0.000308	0.001542
	norfloxacin	11400 [12]	0.000076	0.000382
	ofloxacin	5700 [12]	0.000492	0.002464
	pefloxacin	11428 [14]	0.000225	0.001125
Macrolides	azithromycin	1700 [12]	0.000541	0.002702
	clarithromycin	200 [12]	0.005644	0.028299
	roxithromycin	400 [12]	N.D.	N.D.
NSAIDs	naproxen	7100 [11]	N.D.	N.D.
Sulfonamides and Trimethoprim	sulfacetamide	10000 [10]	N.D.	N.D.
	sulfadimethoxine	10000 [12]	N.D.	N.D.
	sulfamethoxazole	5714 [11]	N.D.	N.D.
	sulfapyridine	3571 [11]	N.D.	N.D.
	trimethoprim	4000 [12]	0.000308	0.001549
Tetracyclines	tetracycline	5714 [11]	0.000092	0.000464
**Hazard Indexes:**			0.01744	0.087359

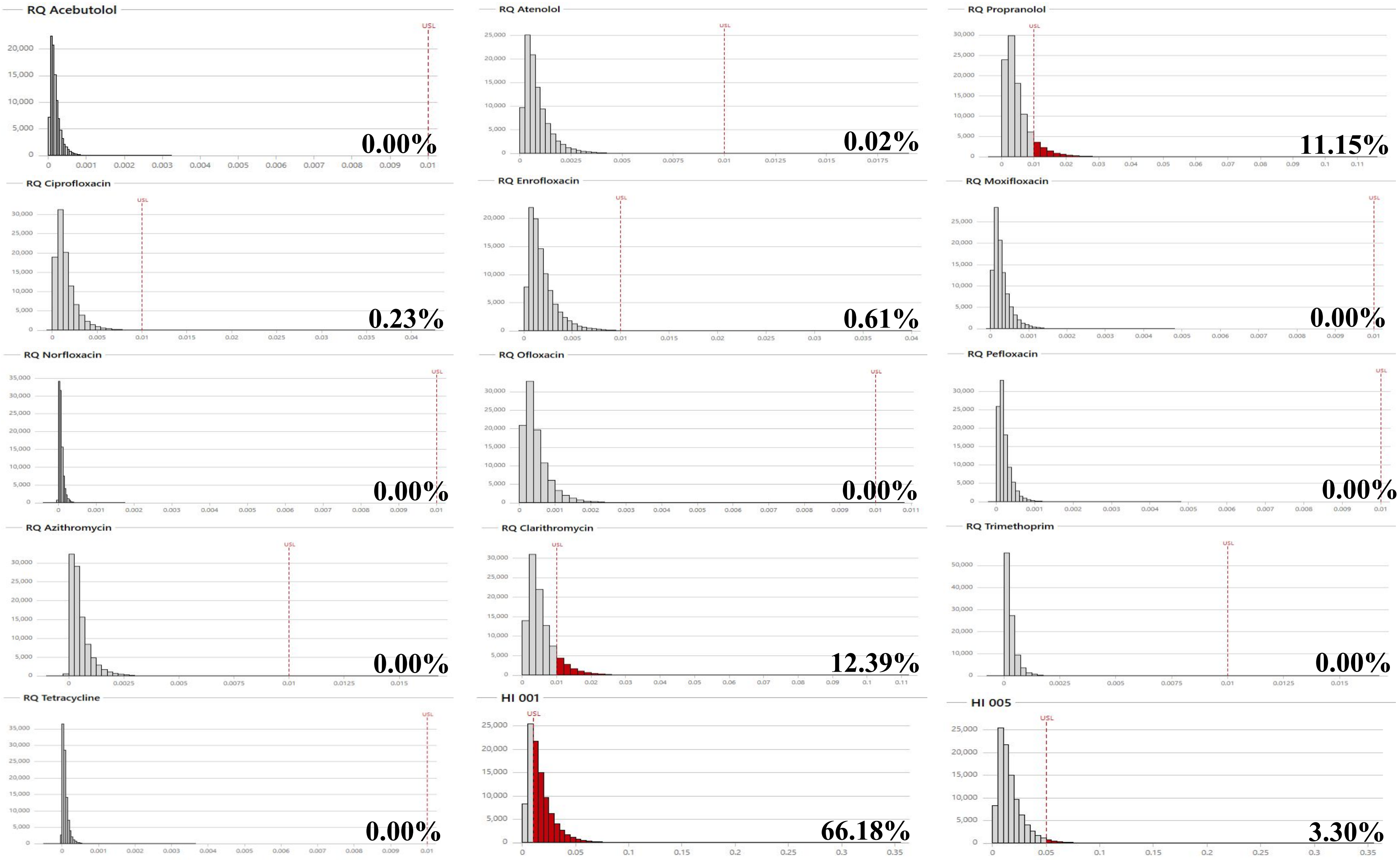

Figure S14. Cumulative distribution of Risk Quotients (RQs) and Hazard Indexes (HIs) of pharmaceuticals at a water concentration of 10 μg·L⁻¹ for adults. The Y-axis represents frequency, and the X-axis denotes RQ or HI values. Percentage values indicate the probability of exceeding an RQ (or HI) value of 0.01, except in the final figure, where a threshold of 0.05 is used.

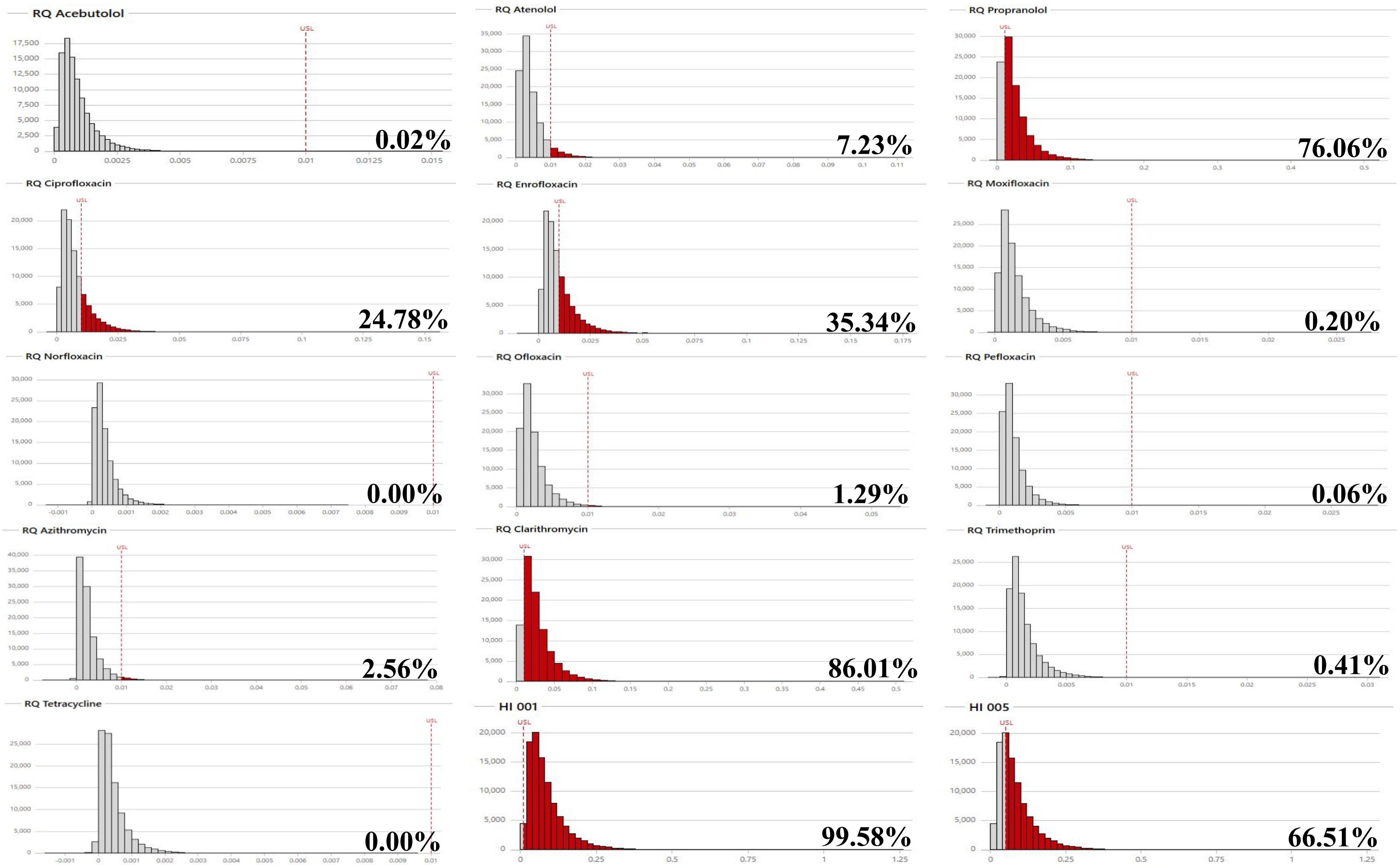

Figure S15. Cumulative distribution of Risk Quotients (RQs) and Hazard Indexes (HIs) of pharmaceuticals at a water concentration of 50 μg·L⁻¹ for adults. The Y-axis represents frequency, and the X-axis denotes RQ or HI values. Percentage values indicate the probability of exceeding an RQ (or HI) value of 0.01, except in the final figure, where a threshold of 0.05 is used.

Appendix 4. Calculation Procedure to Estimate Potential Risk towards Antimicrobial Resistance

The risk quotients (RQs) towards the emergence of antimicrobial resistance in the water environment were assessed at day 0 and after 35 days for various pharmaceutical concentrations under hydroponic conditions. Specifically, water (hydroponic solutions) were spiked at concentrations of 10 and 50 $\mu g \cdot L^{-1}$.

PNEC-MIC (Predicted No-Effect Concentration - Minimum Inhibitory Concentration) water values were sourced from study[18], providing insights into the potential risk of antimicrobial resistance in aquatic environments (Table S8). RQ values were determined as the ratio of measured environmental concentrations (MEC) to predicted no-effect concentrations (PNEC). Furthermore, to account for the co-occurrence of multiple veterinary antimicrobials in water, we calculated the cumulative risk quotient (∑RQ) to assess the comprehensive ecological risks associated with antimicrobial resistance.[19] The criteria for interpreting RQs were as commonly used: low risk when RQ < 0.1, medium risk when 0.1 < RQ < 1, and high risk for RQ > 1.[20,21]

Table S8. Risk Quotients towards Emergence of Antimicrobial Resistance (PNEC-MIC water values were obtained from study[18])

Pharmaceutical Group	Pharmaceutical Name	PNEC-MIC water [$\mu g \cdot L^{-1}$]	Range of Risk Quotients towards Emergence of Antimicrobial Resistance			
			Initial Hydroponic Water Contamination 10 $\mu g \cdot L^{-1}$		Initial Hydroponic Water Contamination 50 $\mu g \cdot L^{-1}$	
			0 day	35 days	0 day	35 days
fluoroquinolones	ciprofloxacin	0.06	167	51	833	254
	enrofloxacin	0.06	167	60	833	302
	moxifloxacin	0.13	77	32	385	160
	norfloxacin	0.5	20	6.3	100	32
	ofloxacin	0.5	20	11	100	55
	pefloxacin	8	1.3	0.6	6.3	2.9
macrolides	azithromycin	0.25	40	23	200	117
	clarithromycin	0.25	40	13	200	65
	roxithromycin	1	10	5	50	25
sulfonamides	sulfacetamide	N.D.	N.D.	N.D.	N.D.	N.D.
	sulfadimethoxine	N.D.	N.D.	N.D.	N.D.	N.D.
	sulfamethoxazole	16	0.6	0.1	3.1	0.6
	sulfapyridine	N.D.	N.D.	N.D.	N.D.	N.D.
	trimethoprim	0.5	20	11	100	57
tetracyclines	tetracycline	1	10	5.8	50	29
**Accumulative Risk Quotients**		**∑RQ**	572	220	2,861	1,099

REFERENCES

(1) NEIL S. MATTSON; CARI PETERS. A Recipe for Hydroponic Success. *Inside Grower*. January 2014, pp 16–19.

(2) Kim, S.; Chen, J.; Cheng, T.; Gindulyte, A.; He, J.; He, S.; Li, Q.; Shoemaker, B. A.; Thiessen, P. A.; Yu, B.; Zaslavsky, L.; Zhang, J.; Bolton, E. E. PubChem 2023 Update. *Nucleic Acids Res* 2023, *51* (D1), D1373–D1380. https://doi.org/10.1093/nar/gkac956.

(3) Knox, C.; Wilson, M.; Klinger, C. M.; Franklin, M.; Oler, E.; Wilson, A.; Pon, A.; Cox, J.; Chin, N. E. (Lucy); Strawbridge, S. A.; Garcia-Patino, M.; Kruger, R.; Sivakumaran, A.; Sanford, S.; Doshi, R.; Khetarpal, N.; Fatokun, O.; Doucet, D.; Zubkowski, A.; Rayat, D. Y.; Jackson, H.; Harford, K.; Anjum, A.; Zakir, M.; Wang, F.; Tian, S.; Lee, B.; Liigand, J.; Peters, H.; Wang, R. Q. (Rachel); Nguyen, T.; So, D.; Sharp, M.; da Silva, R.; Gabriel, C.; Scantlebury, J.; Jasinski, M.; Ackerman, D.; Jewison, T.; Sajed, T.; Gautam, V.; Wishart, D. S. DrugBank 6.0: The DrugBank Knowledgebase for 2024. *Nucleic Acids Res* 2024, *52* (D1), D1265–D1275. https://doi.org/10.1093/nar/gkad976.

(4) Mravcová, L.; Amrichová, A.; Navrkalová, J.; Hamplová, M.; Sedlář, M.; Gargošová, H. Z.; Fučík, J. Optimization and Validation of Multiresidual Extraction Methods for Pharmaceuticals in Soil, Lettuce, and Earthworms. *Environmental Science and Pollution Research* 2024. https://doi.org/10.1007/s11356-024-33492-7.

(5) Chong, J.; Soufan, O.; Li, C.; Caraus, I.; Li, S.; Bourque, G.; Wishart, D. S.; Xia, J. MetaboAnalyst 4.0: Towards More Transparent and Integrative Metabolomics Analysis. *Nucleic Acids Res* 2018, *46* (W1), W486–W494. https://doi.org/10.1093/nar/gky310.

(6) Tang, D.; Chen, M.; Huang, X.; Zhang, G.; Zeng, L.; Zhang, G.; Wu, S.; Wang, Y. SRplot: A Free Online Platform for Data Visualization and Graphing. *PLoS One* 2023, *18* (11), e0294236. https://doi.org/10.1371/journal.pone.0294236.

(7) Geng, J.; Liu, X.; Wang, J.; Li, S. Accumulation and Risk Assessment of Antibiotics in Edible Plants Grown in Contaminated Farmlands: A Review. *Science of The Total Environment* 2022, *853*, 158616. https://doi.org/10.1016/j.scitotenv.2022.158616.

(8) Wu, X.; Ernst, F.; Conkle, J. L.; Gan, J. Comparative Uptake and Translocation of Pharmaceutical and Personal Care Products (PPCPs) by Common Vegetables. *Environ Int* 2013, *60*, 15–22. https://doi.org/10.1016/j.envint.2013.07.015.

(9) EPA, U. Exposure Factors Handbook 2011 Edition (Final). *Washington, DC* 2011, *414*.

(10) Khan, U.; Nicell, J. Human Health Relevance of Pharmaceutically Active Compounds in Drinking Water. *AAPS J* 2015, *17* (3), 558–585. https://doi.org/10.1208/s12248-015-9729-5.

(11) Prosser, R. S.; Sibley, P. K. Human Health Risk Assessment of Pharmaceuticals and Personal Care Products in Plant Tissue Due to Biosolids and Manure Amendments, and Wastewater Irrigation. *Environ Int* 2015, *75*, 223–233. https://doi.org/10.1016/j.envint.2014.11.020.

(12) Liu, S.; Zhao, H.; Lehmler, H.-J.; Cai, X.; Chen, J. Antibiotic Pollution in Marine Food Webs in Laizhou Bay, North China: Trophodynamics and Human Exposure Implication. *Environ Sci Technol* 2017, *51* (4), 2392–2400. https://doi.org/10.1021/acs.est.6b04556.

(13) Ji, K.; Kho, Y.; Park, C.; Paek, D.; Ryu, P.; Paek, D.; Kim, M.; Kim, P.; Choi, K. Influence of Water and Food Consumption on Inadvertent Antibiotics Intake among General Population. *Environ Res* 2010, *110* (7), 641–649. https://doi.org/10.1016/j.envres.2010.06.008.

(14) Sengar, A.; Vijayanandan, A. Human Health and Ecological Risk Assessment of 98 Pharmaceuticals and Personal Care Products (PPCPs) Detected in Indian Surface and Wastewaters. *Science of The Total Environment* 2022, *807*, 150677. https://doi.org/10.1016/j.scitotenv.2021.150677.

(15) Keerthanan, S.; Jayasinghe, C.; Biswas, J. K.; Vithanage, M. Pharmaceutical and Personal Care Products (PPCPs) in the Environment: Plant Uptake, Translocation, Bioaccumulation, and Human Health Risks. *Crit Rev Environ Sci Technol* 2021, *51* (12), 1221–1258. https://doi.org/10.1080/10643389.2020.1753634.

(16) Moazeni, M.; Heidari, Z.; Golipour, S.; Ghaisari, L.; Sillanpää, M.; Ebrahimi, A. Dietary Intake and Health Risk Assessment of Nitrate, Nitrite, and Nitrosamines: A Bayesian Analysis and Monte Carlo Simulation. *Environmental Science and Pollution Research* 2020, *27* (36), 45568–45580. https://doi.org/10.1007/s11356-020-10494-9.

(17) Sanaei, F.; Amin, M. M.; Alavijeh, Z. P.; Esfahani, R. A.; Sadeghi, M.; Bandarrig, N. S.; Fatehizadeh, A.; Taheri, E.; Rezakazemi, M. Health Risk Assessment of Potentially Toxic Elements Intake via Food Crops Consumption: Monte Carlo Simulation-Based Probabilistic and Heavy Metal Pollution Index. *Environmental Science and Pollution Research* 2021, *28* (2), 1479–1490. https://doi.org/10.1007/s11356-020-10450-7.

(18) AMR Industry Alliance. *AMR Alliance Science-Based PNEC Targets for Risk Assessments*; 2023.

(19) Fang, L.; Chen, C.; Zhang, F.; Ali, E. F.; Sarkar, B.; Rinklebe, J.; Shaheen, S. M.; Chen, X.; Xiao, R. Occurrence Profiling and Environmental Risk Assessment of Veterinary Antibiotics in Vegetable Soils at Chongqing Region, China. *Environ Res* 2023, *227*, 115799. https://doi.org/10.1016/j.envres.2023.115799.

(20) Bourdat-Deschamps, M.; Leang, S.; Bernet, N.; Daudin, J.-J.; Nélieu, S. Multi-Residue Analysis of Pharmaceuticals in Aqueous Environmental Samples by Online Solid-Phase Extraction–Ultra-High-Performance Liquid Chromatography-Tandem Mass Spectrometry: Optimisation and Matrix Effects Reduction by Quick, Easy, Cheap, Effective, Rugged and Safe Extraction. *J Chromatogr A* 2014, *1349*, 11–23. https://doi.org/10.1016/j.chroma.2014.05.006.

(21) Sun, J.; Zeng, Q.; Tsang, D. C. W.; Zhu, L. Z.; Li, X. D. Antibiotics in the Agricultural Soils from the Yangtze River Delta, China. *Chemosphere* 2017, *189*, 301–308. https://doi.org/10.1016/j.chemosphere.2017.09.040.
